# Genome-Wide Association Study of Circadian Behavior in *Drosophila melanogaster*

**DOI:** 10.1007/s10519-018-9932-0

**Published:** 2018-10-19

**Authors:** Susan T. Harbison, Shailesh Kumar, Wen Huang, Lenovia J. McCoy, Kirklin R. Smith, Trudy F. C. Mackay

**Affiliations:** 1 Department of Biological Sciences, North Carolina State University, Raleigh, NC USA; 2 Laboratory of Systems Genetics, National Heart Lung and Blood Institute, National Institutes of Health, Bethesda, MD USA; 3 Genetics Program and W. M. Keck Center for Behavioral Biology, North Carolina State University, Raleigh, NC USA; 4 Present Address: Department of Animal Science, Michigan State University, East Lansing, MI USA; 5 Laboratory of Systems Genetics, National Heart Lung and Blood Institute, Building 10, Room 7D13, 10 Center Drive, Bethesda, MD 20892-1640 USA; 6 Present Address: Center for Human Genetics and Department of Genetics and Biochemistry, Clemson University, 114 Gregor Mendel Circle, Greenwood, SC 29646 USA

**Keywords:** *Drosophila melanogaster*, Circadian rhythms, Genome-wide association, Period, Rhythmicity index

## Abstract

Circadian rhythms influence physiological processes from sleep–wake cycles to body temperature and are controlled by highly conserved cycling molecules. Although the mechanistic basis of the circadian clock has been known for decades, the extent to which circadian rhythms vary in nature and the underlying genetic basis for that variation is not well understood. We measured circadian period (Ʈ) and rhythmicity index in the *Drosophila* Genetic Reference Panel (DGRP) and observed extensive genetic variation in both. Seven DGRP lines had sexually dimorphic arrhythmicity and one line had an exceptionally long Ʈ. Genome-wide analyses identified 584 polymorphisms in 268 genes. We observed differences among transcripts for nine genes predicted to interact among themselves and canonical clock genes in the long period line and a control. Mutations/RNAi knockdown targeting these genes also affected circadian behavior. Our observations reveal that complex genetic interactions influence high levels of variation in circadian phenotypes.

**Electronic supplementary material:**

The online version of this article (10.1007/s10519-018-9932-0) contains supplementary material, which is available to authorized users.

## Background

Circadian rhythms are endogenous cycles present in almost all living organisms. They affect myriads of biological processes in humans, such as sleep/wake cycles, body temperature, hormone levels, heart rate, and even cognitive performance (Van Dongen et al. 2004). Circadian rhythms may thus play a fundamental role in human health (Zee et al. 2014). Disruption to circadian rhythms has been associated with detrimental neurobehavioral consequences. For example, rotating shift workers, people experiencing chronic jet lag, and those living in areas with extreme long or short photoperiods have increased risk of psychiatric and mood disorders (Bunney and Bunney 2000; Boivin 2000; Grandin et al. 2006; Magnusson and Boivin 2003). Furthermore, circadian disruptions often precede the development of neurodegenerative disorders such as Alzheimer’s disease and Parkinson’s disease, though whether they have a causal role in these conditions is unknown (Mattis and Sehgal 2016; Videnovic and Zee 2015). Disruption of circadian rhythms may also contribute to metabolic disease. For instance, circadian misalignment, often manifested as rotating or shifting work schedules, has been associated with increased hypertension, Type 2 diabetes, total cholesterol, and cardiovascular disease (Reutrakul and Knutson 2015; McHill and Wright 2017; Reutrakul and Van Cauter 2014). Profound effects on metabolic processes such as glucose tolerance, body mass index (BMI) and cortisol levels result from the disruption of sleep and circadian cycles combined with poor dietary choices (Shi and Zheng 2013; McHill et al. 2014). Thus, disrupted circadian rhythms are an important consideration in the etiology of disease.

The circadian period in humans is tightly regulated and close to 24 h (Czeisler et al. 1999). Human clocks have a built-in mechanism to adjust endogenous period and phase in response to a moderate light stimulus (Scheer et al. 2007). Human studies focus on chronotype, which is the preference for morning or evening activity (Kalmbach et al. 2016). Chronotype has been shown to have a genetic component (Jones et al. 2016; Lane et al. 2016; Kalmbach et al. 2016; Toomey et al. 2015). Candidate gene studies show associations between core canonical clock genes and chronotype (Kalmbach et al. 2016). For instance, a hereditary form of delayed sleep phase disorder (DSPD) is associated with a dominant coding variation in the core circadian clock gene *CRY1* (Patke et al. 2017). Recently, three large-scale genome-wide association studies in humans revealed that, in addition to canonical clock genes, novel genes may contribute to differences in human chronotype (Jones et al. 2016; Lane et al. 2016; Hu et al. 2016).

Core clock mechanisms regulating circadian behavior are remarkably similar between mammals and *Drosophila melanogaster*, even though they diverged approximately 600 million years ago (Yu and Hardin 2006; Panda et al. 2002; Nitta et al. 2015). In fact, the genes and processes involved in complex mammalian circadian rhythms were first identified in flies (Konopka and Benzer 1971; Hardin et al. 1990). Rhythmic rest and activity behavior in *Drosophila* is one of the most reliable phenotypes for the identification of novel genes regulating circadian rhythms (Hardin 2011; Helfrich-Forster et al. 2011). The use of flies for rest and activity studies offers several advantages over mammalian models. For example, the availability of an extensive collection of stocks with mapped mutations, chromosomal deletions, and transgenic constructs have facilitated forward genetics approaches in circadian rhythm studies. This approach has identified numerous genes of the circadian clock (Hardin et al. 1990; Takahashi 1993; Reppert and Weaver 2002; Sehgal et al. 1995; Dunlap et al. 2007).

Variability in circadian period and rhythmicity parameters among wild-derived isochromosomal lines of flies has previously been noted (Emery et al. 1994, 1995), and this variation has a genetic component (Emery et al. 1995). Heritability has been estimated at 0.14 for circadian period (Emery et al. 1995). Therefore, naturally occurring polymorphisms likely influence the fly’s circadian clock. Historically, most work has focused on polymorphic variation within the canonical clock genes in natural populations. Studies of a threonine-glycine-encoding repeat region in the *period* gene revealed a clinal distribution of these alleles in natural populations in Europe and Australia (Kyriacou et al. 2008; Sawyer et al. 1997). Likewise, an insertion in the 5′ coding region of *timeless* varies with latitude in European flies (Kyriacou et al. 2008). Furthermore, a polymorphic variant in the genomic region containing *shaggy* is clinal in North America, though not in Australia (Rand et al. 2010). In contrast, a non-synonymous polymorphism in *cryptochrome* does not vary with latitude but persists in nearly equal frequencies in European populations (Pegoraro et al. 2014). These polymorphisms are associated with differences in temperature-compensated period length (Kyriacou et al. 2008; Sawyer et al. 1997), the frequency of diapause (Kyriacou et al. 2008; Tauber et al. 2007), the timing of eclosion (Pegoraro et al. 2014), and thermal tolerance (Rand et al. 2010; Sawyer et al. 1997). These experiments demonstrate the importance of polymorphic variation in canonical clock genes to circadian behavior in flies; however, the contribution of polymorphic variants in the rest of the genome to circadian behavior in natural populations remains largely unexplored.

Recently, several efforts have been made to study genomic differences for a variety of behaviors, diseases and life history traits using genetic reference populations of *Drosophila* (King et al. 2012a, b; Mackay et al. 2012; Huang et al. 2014; Grenier et al. 2015). Here, we performed a genome-wide association study (GWAS) using the *D. melanogaster* Genetic Reference Panel (DGRP), a unique resource created by mating full siblings of wild-caught iso-female lines for 20 generations (Mackay et al. 2012; Huang et al. 2014), to explore the range of polymorphic variants contributing to genetic variation in *Drosophila* circadian phenotypes. Some of the salient features of the DGRP collection are (i) availability of full sequence data, (ii) rapid decay in linkage disequilibrium (LD) with physical distance, (iii) and a lack of population structure (Mackay et al. 2012; Huang et al. 2014). Another important feature of the DGRP is the ability to perform screening of identical genotypes in a controlled environment, thus elucidating the role of genes in micro-environmental plasticity (Morgante et al. 2015; Lin et al. 2016a). Many studies ranging from natural variation in physiological processes (food intake and nutrient stores) (Garlapow et al. 2015; Unkless et al. 2015), to variation in behavior (olfactory avoidance and aggression) (Arya et al. 2015; Shorter et al. 2015), to anatomical features (mushroom body size and abdominal pigmentation) (Dembeck et al. 2015; Zwarts et al. 2015), and to environmental and drug responses (radiation resistance and paraquat) (Vaisnav et al. 2014; Weber et al. 2012) have exploited the DGRP.

In this study, we examined two circadian phenotypes, circadian period (Ʈ) and rhythmicity index (RI), a measure of how consistent the fly’s daily activity patterns are, in 167 DGRP lines. We observed high levels of sex-specific genetic variation for both Ʈ and RI. Several lines had sex-specific arrhythmic behavior and one line (DGRP_892) had a very long average circadian period of 31 h. The GWAS identified 584 molecular polymorphisms in 268 candidate genes associated with Ʈ and RI. Thus, the association mapping strategy enabled us to identify candidate genes affecting variation in circadian behavior.

## Results

### Quantitative genetic analyses

**Fig. 1:**
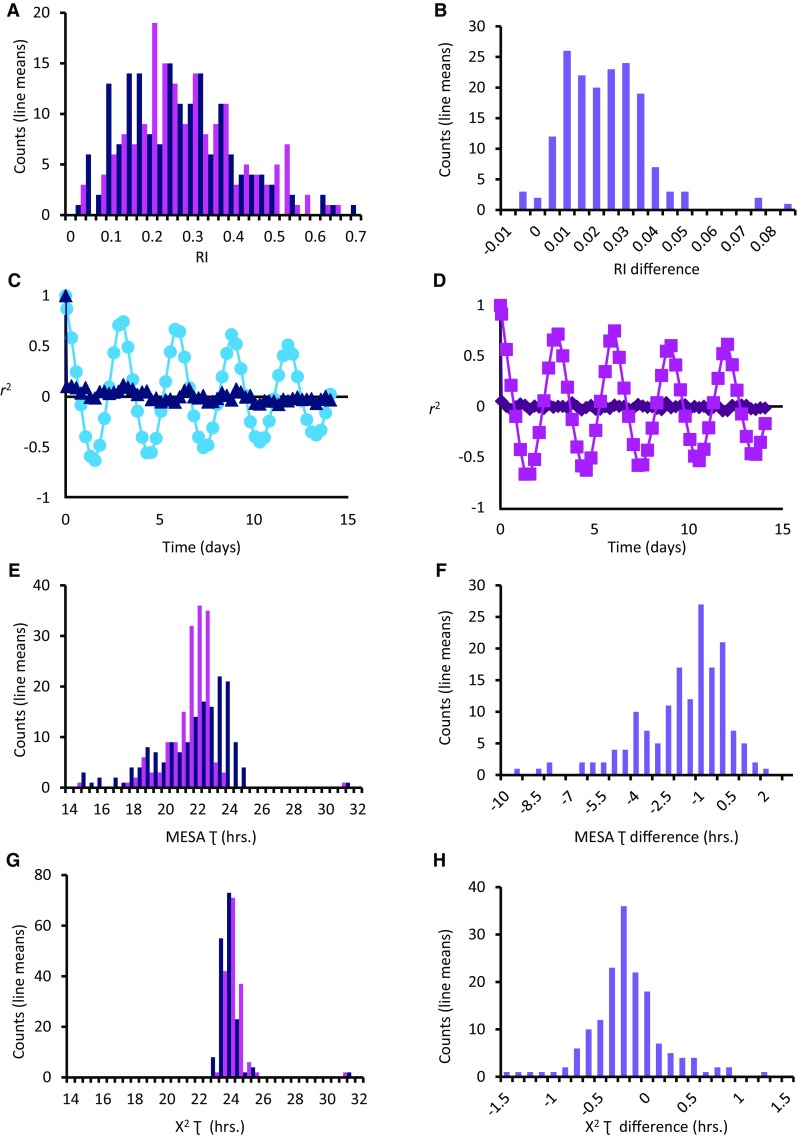
Distribution of circadian rhythm phenotypes in the DGRP. Male line means are shown in dark blue; female line means are shown in pink. The difference in line means (male–female) is shown by purple bars. **a** Rhythmicity index. **b** Difference in rhythmicity index. **c** Autocorrelation plot for a representative male of a rhythmic line (DGRP_383; RI = 0.667) and an arrhythmic line (DGRP_101; RI = 0.066). **d** Autocorrelation plot for a representative female of a rhythmic line (DGRP_861; RI = 0.748) and an arrhythmic line (DGRP_375; RI = 0.037). **e** MESA period. **f** Difference in MESA period. **g** χ^2^ period. **h** Difference in χ^2^ period

Circadian phenotypes were highly variable in the DGRP. We observed high levels of genetic variation among lines (all *P*_Line(Block)_ < 0.0001, four-way nested ANOVA) for RI and for Ʈ, whether Ʈ was calculated using the Maximum Entropy Spectral Analysis method (MESA period) or by the Chi square Periodogram method (χ^2^ period) (Fig. 1; Table S1). These traits were also highly sexually dimorphic among lines (all *P*_Sex×Line(Block)_ < 0.001, four-way nested ANOVA). Broad-sense heritability was high for RI (*H*^2^ = 0.43), indicating that the genetic contribution to this trait is relatively large (Fig. 1a). Heritability was relatively low for MESA period (*H*^2^ = 0.17), consistent with previous estimates of heritability in wild-derived populations (Emery et al. 1995), but higher for χ^2^ period (*H*^2^ = 0.39). We observed that ~ 12% of the flies were arrhythmic, a figure consistent with previous reports of rhythmicity in natural populations (Emery et al. 1994; Kumar et al. 2005). Females tended to have a lower RI than males and were more likely than males to be arrhythmic (Fig. 1b). Female RI averages for lines DGRP_42, DGRP_153, DGRP_375, DGRP_509, DGRP_810, and DGRP_908 were below the threshold value for rhythmicity (${r_k}$ = 0.0772). Females from these lines were therefore classified as arrhythmic. Males of one line, DGRP_101, were also classified as arrhythmic. Representative examples of autocorrelation plots for rhythmic and arrhythmic flies are shown in Fig. 1c, d. The distributions of MESA and χ^2^ period across the DGRP were quite different from one another (Fig. 1e–h). The range of values for the period as calculated by Chi square periodogram was smaller than the range of values seen using MESA, particularly on the low end of the scale. Ʈ estimates of line means varied from 15 to 31.3 h when calculated using MESA and from 23.2 to 31.3 h when calculated using the χ^2^ periodogram method. Interestingly, both methods identified a single line with a very long period, line DGRP_892. This line had an average period of 31.76 h in males and 30.92 h in females as calculated by the MESA and 31.78 (30.88) hours as calculated by χ^2^ for males (females) (Table S2; for a representative actogram, see Fig. S1A). DGRP_892 had long sleep in LD but was not an outlier (Harbison et al. 2013), nor was there a phase shift in the activity of these flies under LD conditions (Fig. S1B). Overall, circadian phenotypes were both variable and heritable in this natural population of flies.

We calculated the genetic correlations among all circadian parameters (Table S3). It has been noted previously that rhythmicity index does not predict circadian period; rather, it is an indicator of strength or robustness of the circadian rhythm (Grierson et al. 2016; Dowse 2007). Not surprisingly, the genetic correlation *r*_G_ between RI and Ʈ was also low, though statistically significant, in the DGRP: *r*_G_ was only 0.18 between RI and MESA period, and − 0.15 between RI and χ^2^ period. Thus, we expect that few genes will be common to RI and Ʈ. The genetic correlation between MESA period and χ^2^ period was 0.71 if line DGRP_892 was included, and 0.61 without this line. A perfect overlap between the two period calculations would not be expected as the distributions of the two traits are quite distinct; many more estimates of period occur in the low range for MESA period than for χ^2^ period (Fig. 1e, g; see also “Discussion”). These correlations suggest that some, but not all genes will be common among circadian traits.

Sleep and circadian rhythm behaviors are related as the timing of sleep is regulated by the circadian clock (Borbely 1982). We therefore correlated the circadian rhythm phenotypes with sleep phenotypes measured on the DGRP in a previous study (Harbison et al. 2013). The sleep study measured duration (minutes spent sleeping), bout number (number of naps), and average bout length (average nap length) during the day and night. It also measured waking activity, the number of activity counts per minute spent awake. In addition, the study measured the environmental sensitivity (coefficient of environmental variation, or *CV*_*E*_) (Mackay and Lyman 2005) for each sleep and activity trait (Harbison et al. 2013). Both MESA and χ^2^ period had significant genetic correlations with night average bout length (*r*_G_ = 0.27 and 0.28, respectively). However, only MESA period was significantly genetically correlated with night bout number (*r*_G_ = − 0.25) and night bout number *CV*_E_ (*r*_G_ = 0.41) (Table S4). RI had significant genetic correlations night and day bout number (*r*_G_ = − 0.32 and − 0.52, respectively), night average bout length (*r*_G_ = 0.32), night and day bout number *CV*_E_ (*r*_G_ = 0.37 and 0.28, respectively), and day average bout length *CV*_E_ (*r*_G_ = 0.26) (Table S4). While the genetic correlations between sleep and circadian phenotypes were statistically significant, inspection of Table S4 reveals that the correlations are not high. In addition, we computed sleep phenotypes from the rest and activity data collected in constant darkness in this study and used the data to calculate the phenotypic and genetic correlations with the circadian rhythm parameters. For RI, significant correlations were observed in the same traits (day and night bout number, and night average bout length), but the magnitudes of the correlations were higher (Table S4). A few additional sleep traits were correlated with the circadian rhythm phenotypes: day sleep duration and waking activity with RI, night bout number with MESA, and day average bout length and day sleep with χ^2^ period. Therefore, some common genetic architecture exists between sleep and circadian phenotypes, but it is far from being a complete overlap.

### Genotype–phenotype associations

The DGRP is fully sequenced (Mackay et al. 2012; Huang et al. 2014), and all single nucleotide polymorphisms (SNPs), non-SNP variants (indels, tandem duplications, and complex variants), and chromosomal inversions have been mapped (Huang et al. 2014). We used the DGRP2 web-based analysis to associate these 1,920,276 genetic variants with the circadian phenotypes (Table S5) (Huang et al. 2014). Circadian phenotypes were not impacted by *Wolbachia* infection status, but some phenotypes were significantly associated with chromosomal inversions. RI in males was associated with *In(2R)NS* and *In(3R)Mo*; combined-sex RI was associated with *In(2R)NS*. In addition, the unusually long period observed in DGRP_892 was likely to impact the results of genome-wide association on circadian period as its inclusion would violate the assumption of independently and identically distributed residuals (Mackay et al. 2012). We therefore conducted the genome-wide analyses for circadian period without DGRP_892. We found 142 significant polymorphisms in 73 genes for RI; and 292 polymorphisms in 139 genes for MESA and 150 in 65 genes for χ^2^ period (Table S5) at a threshold *P*-value of 1 × 10^−5^. This threshold *P*-value is consistent with previous studies of quantitative traits in the DGRP (Arya et al. 2015; Dembeck et al. 2015a, b; Garlapow et al. 2015; Hunter et al. 2016; Shorter et al. 2015; Zwarts et al. 2015). Quantile–quantile (Q–Q) plots showed an excess of *P*-values below 10^−5^ for all traits, suggesting that variants below this threshold are enriched for true positive associations (Fig. S2–S4). At this threshold, the false-discovery rates range from 0.0092 to 0.52 for RI, 0.0033–0.25 for MESA period; and < 0.0001–0.28 for χ^2^ period (Table S5). A total of thirty-nine variants had *P*-values that were less than a Bonferroni-corrected *P*-value of 1.6 × 10^− 8^. Variants associated with larger effect sizes had lower minor allele frequencies, consistent with previous observations of complex traits in the DGRP (Fig. S5) (Jordan et al. 2012; Weber et al. 2012). We hypothesized that polymorphisms lying within a gene or ± 1000 bp from a gene affect that gene as linkage disequilibrium decays over an average 30–200 bp in the DGRP (Mackay et al. 2012).

**Fig. 2:**
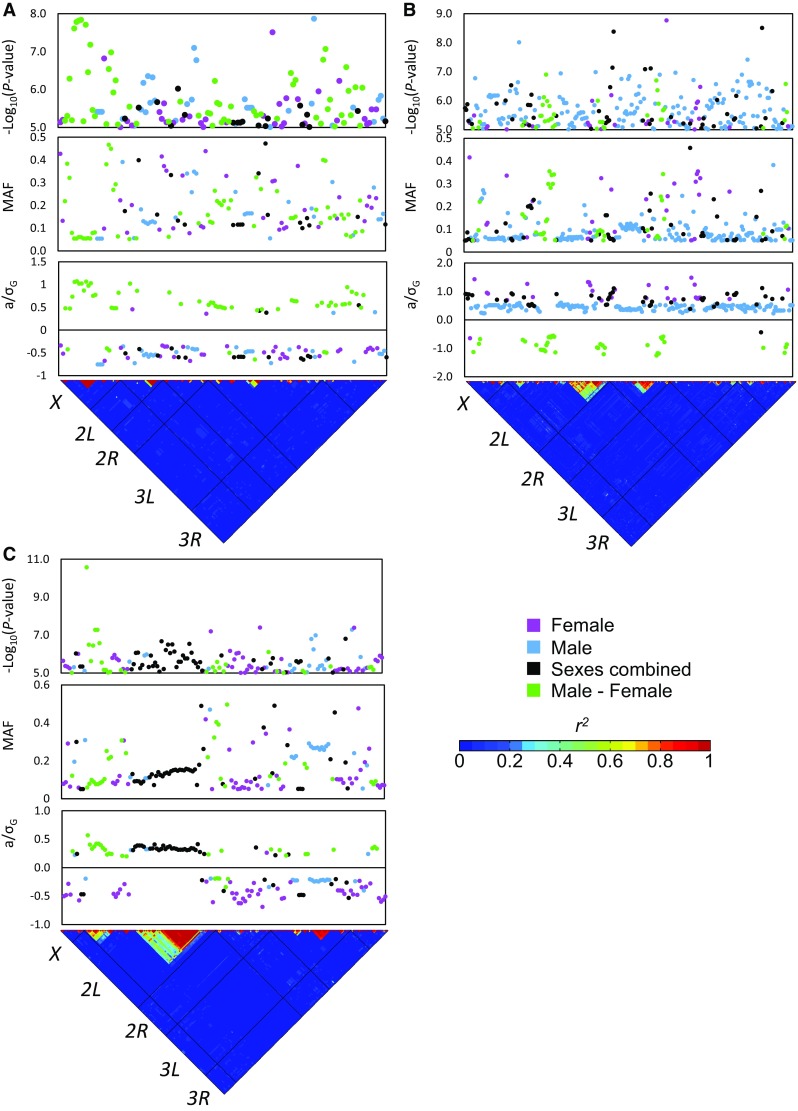
Genome-wide association results for circadian rhythm phenotypes. Polymorphisms with *P*-values for association ≤ 1 × 10^−5^ are plotted. The top panel shows the *P*-values plotted as − log_10_(*P*-value). The middle panel shows the minor allele frequency (MAF) of each variant in the DGRP; only variants with a MAF of 0.05 or greater were considered in the analysis. Effect sizes are plotted as *a*/*σ*_G_ in the bottom panel, where *σ*_G_ is the among-line standard deviation. The triangle shows the linkage disequilibrium among significant variants as *r*^2^, in chromosomal order. **a** Rhythmicity index. **b** MESA period. **c** χ^2^ period

Plots of significant variants for each trait plotted against minor allele frequency, − log_10_(*P*-value), and normalized effect size reveal the complex genetic architecture of circadian behavior. Both low-frequency and common polymorphisms affect RI (Fig. 2a and S4). Effect sizes were relatively small, and virtually no linkage disequilibrium was observed between the top variants. In contrast, significant polymorphisms for period tended to have low minor allele frequencies, higher *P*-values, and larger effect sizes (Fig. 2b, c). Some linkage disequilibrium is present on chromosomes *2L* and *2R* among variants associated with MESA period, and on chromosomes *X* and *2L* among variants associated with χ^2^ period. Five genes overlapped between the two period measures: *CG31676, Mob2, nAcRalpha-30D, Oamb*, and *shep*. Variants in canonical clock genes were not implicated in circadian period, but a single intronic SNP in *Pdp1* was significantly associated with RI. Several other candidate genes or their homologs in other species have known or predicted effects on circadian behavior: *5-HT1A* (Yuan et al. 2006), *CG14618* (Ueda et al. 2002), *CG42321* (Abruzzi et al. 2011), *Gga* (Hughes et al. 2010), *Rh5* (Szular et al. 2012), *slo* (Ceriani et al. 2002; Fernandez et al. 2007), *Tep4* (Hughes et al. 2010; Hu et al. 2016), *tinc* (Mizrak et al. 2012), *tnc* (Hughes et al. 2010), and *wap* (Wu et al. 2008). Overall, we identified 584 unique polymorphisms that implicated 268 genes in circadian behavior.

### Epistatic associations

To search for epistatic interactions between pairs of variants that are associated with the traits, we performed a full genome-wide pair-wise search for all variants with a minor allele frequency (MAF) greater than 0.15. Variants in linkage disequilibrium (*r*^2^ ≥ 0.8) were pruned. We found 2,295,186 epistatic interactions for circadian period at a nominal *P*-value ≤ 1 × 10^− 7^ or less. For χ^2^ period, there were 285,470 interactions for males and 38,411 for females. For MESA period, there were 100,701 interactions for males and 1,960,053 for females. The large differences in the number of interactions found for the two measures of circadian period are likely due to the differences in their distribution; χ^2^ period had a much lower range of values for period than MESA, particularly for the lower values of circadian period (Fig. 1). For RI, there were 29,465 interactions for males and 23,422 interactions for females. A total of 6401 unique epistatic interactions for χ^2^ and MESA period were within or within ± 1 kb of the core circadian clock genes: *per, tim, Clk, cyc, Pdp1, vri*, and *sgg* (Table S6). These previously unappreciated interactions suggested that circadian period could be modified by numerous interactions with the molecular circadian clock. Likewise, 189 epistatic interactions for RI contained canonical clock genes (Table S6). Despite pruning the data to a relatively high MAF and accounting for potential linkage disequilibrium, these large numbers of epistatic interactions were likely to have numerous false positives. We therefore followed up a subset of these interactions with further testing (see below).

### Comparison with sleep genome-wide association

The circadian clock is thought to control the timing of sleep (Borbely 1982). We examined the degree of overlap between the variants we identified for circadian behavior with a previous genome-wide association study of sleep (Harbison et al. 2013). Only one variant overlapped between MESA period and a single sleep trait, average day bout length; two variants overlapped between χ^2^ period and waking activity *CV*_E_. However, if we considered the overlap among genes, 48 putative circadian genes overlapped with mean night sleep, night and day average bout length, and waking activity; 100 candidate circadian genes overlapped with measures of environmental sensitivity for night sleep, day sleep, day bout number, night average bout length, and waking activity (Table S7). Two of these genes were implicated in sleep and its regulation in other studies: *Sh* (Cirelli et al. 2005) and *5-HT1A* (Yuan et al. 2006). Thus, while the overlap among polymorphic variants was low, the overlap between candidate genes in the sleep and circadian studies was moderate. Interestingly, of the 4440 genes predicted to interact epistatically with canonical clock genes, 991 genes were also implicated in sleep and waking activity (Harbison et al. 2013), 9.4% more than would be expected by random chance (Table S8).

### Candidate genes potentially contributing to long circadian periods in DGRP_892

None of the significant polymorphisms we identified as associated with circadian period were in coding or known non-coding regulatory regions of the canonical clock genes. Furthermore, none of the DGRP variants predicted to alter coding sequence are found in canonical clock genes (*per, tim, vri, Pdp1, Clk, sgg, cyc*, and *dbt*) (Huang et al. 2014), nor did we find any naturally-occurring polymorphisms known to produce a long circadian period that were private to DGRP_892 (Konopka and Benzer 1971; Kloss et al. 1998). This suggests that the extreme circadian period seen in DGRP_892 may be due to the action of regulatory genes on core clock genes (Ray and Reddy 2016). Polymorphisms within or in close proximity to such regulatory genes may influence the extremely long Ʈ in DGRP_892 by changing transcript abundance (Albert and Kruglyak 2015), mRNA secondary structure (Nackley et al. 2006; Wang et al. 2007), or protein structure (Kimchi-Sarfaty et al. 2007). Thus, we examined transcript abundance over time of three canonical clock genes in DGRP_892: *per, tim*, and *Pdp1*, as well as 26 candidate genes from this study whose transcripts were previously reported to be either cycling and/or regulated by the circadian clock (Table S9). Six of these candidate genes came from the main effect analyses, while the remaining genes came from the epistatic analysis. Genes from the epistatic analysis interacted with either canonical clock genes or other genes identified in the GWA (Table S9). In addition, six candidate genes had intergenic variants proximal to predicted circadian regulatory motifs (i.e., E-boxes) that may influence promoter or terminator activity, messenger RNA (mRNA) conformation (stability), and subcellular localization of mRNAs and/or proteins (Komar et al. 1999; Komar 2007). Transcript levels for each gene were compared to Canton-S B, an isogenic line with a normal circadian period (23.81 ± 0.03 h in males; 24.05 ± 0.18 h in females). If these genes affect period in DGRP_892, we would expect to see differences in the magnitude of gene expression and/or cycling between the two lines.

### Canonical circadian clock transcript levels

We examined transcript levels of *per, tim*, and *Pdp1* at six circadian timepoints under both standard (LD) and constant dark (DD) lighting conditions. We first analyzed the changes in gene expression over time within each line (Table S10). As anticipated, transcripts of *per* were significantly different across time in Canton-S B, whether in DD or LD (FDR = 0.0001 and 0.0127, respectively), with a peak of expression around CT10 in DD and ZT10-14 in LD (post-hoc Tukey *P* ≤ 0.05). Likewise, *tim* expression was significantly different in *Canton-S B* across time for both lighting conditions (FDR = 0.0013 for DD and 0.0273 for LD); expression peaked at CT10 in DD and ZT10-14 in LD (post-hoc Tukey *P* ≤ 0.05). However, *Pdp1* showed marginally non-significant differences in gene expression across time in Canton-S B under both lighting conditions (FDR = 0.0868 in DD and 0.0922 in LD), though there was a significant peak of expression in LD as ZT14 was higher than the other time points (post-hoc Tukey *P* ≤ 0.05). We used the JTK_CYCLE program to determine whether these genes were cycling rhythmically. Both *per* and *tim* cycled robustly in both LD and DD lighting conditions (FDR = 0.0002 and 0.0019 for *per*, respectively, and FDR = 0.0007 and 0.0488 for *tim*, respectively). *Pdp1* exhibited significant cycling only in LD (FDR = 0.001). Thus, Canton-S B had the expected pattern of cycling gene expression for *per* and *tim; Pdp1* appeared slightly damped.

In contrast to the canonical clock transcripts observed in Canton-S B, gene expression patterns differed in DGRP_892 (Table S10). *per* transcriptional cycling appeared damped in DGRP_89*2*, particularly in DD (FDR = 0.0922 in DD and 0.0681 in LD). JTK_CYCLE analysis confirmed that *per* was not cycling in a 24-h period in LD or in DD. In LD, there was a peak of *per* expression at approximately ZT14 (post-hoc Tukey *P* ≤ 0.05). *tim* appeared to cycle robustly in DGRP_892 under both lighting conditions as expression changed across time (FDR = 0.0050 for DD and 0.0273 for LD) with a peak of expression at CT10 in constant darkness (post-hoc Tukey *P* ≤ 0.05). Analysis with JTK_CYCLE confirmed that *tim* had rhythmic expression in LD, while rhythmicity in DD was not formally significant (FDR = 0.0210 and 0.0735, respectively). *Pdp1* transcripts were not differentially expressed across time in DGRP_892 under either lighting condition, but JTK_CYCLE analysis indicated that the transcripts were cycling in LD (*P* = 0.0430). Transcriptional cycling may therefore be impaired in both *per* and *Pdp1* in DGRP_892.

**Fig. 3:**
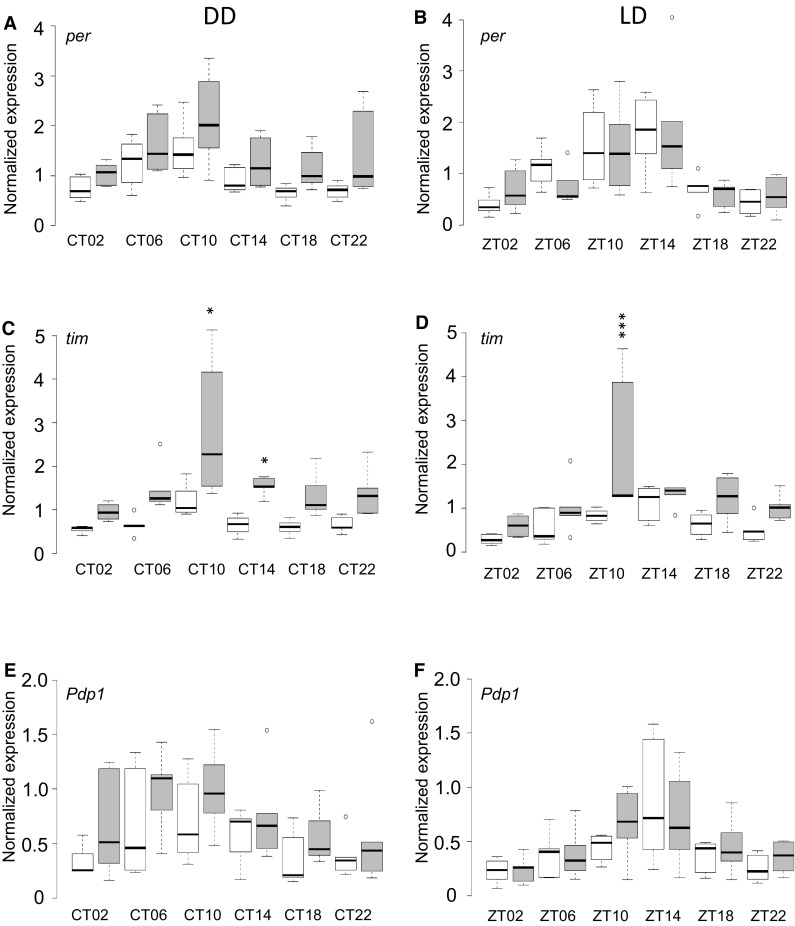
Canonical clock gene expression profile of Canton-S B and DGRP_892. Normalized transcript levels are plotted against time for constant darkness (DD) and standard 12-h light:dark (LD) conditions. Canton-S B transcript levels are white; DGRP_892 transcript levels are gray. **a, b***per*. **c, d***tim*. **e, f***Pdp1. CT* circadian time, *ZT* zeitgeber time. Plots show the average of males and females. Significant differences between Canton-S B and DGRP_892 at a given time point are indicated by asterisks. *FDR < 0.05; ***FDR < 0.001. Source data is provided in Table S12

We next asked whether there were significant differences in gene expression between Canton-S B and DGRP_892 in these canonical clock genes (Table S11). *per* expression was significantly different between the two lines (FDR = 0.0482), with DGRP_892 having higher levels in DD (FDR < 0.0001) (Fig. 3a). Gene expression between the two lines was not significantly different in LD (Fig. 3b). DGRP_892 had higher levels of *tim* than Canton-S B, whether gene expression was measured in LD or in DD (FDR < 0.0001) (Fig. 3c, d). *tim* expression levels in DGRP_892 were much higher than Canton-S B at CT14 in DD (FDR = 0.0387), and at ZT10 in LD (FDR = 0.0019) (Table S11). *Pdp1* levels were higher in DGRP_892 than Canton-S B across time and lighting condition (FDR = 0.0388) (Fig. 3e, f), but the contrast between the two lines was only different in DD (FDR = 0.0360) when times or lighting conditions were considered separately. Given the wealth of reports of differences in transcript abundance in either sex (Jin et al. 2001; Arbeitman et al. 2002; Parisi et al. 2003; Ranz et al. 2003; Harbison et al. 2005; Wayne et al. 2007; Zhang et al. 2007; Ayroles et al. 2009; Huylmans and Parsch 2014; Huang et al. 2015), we expected to see differences in males and females. Only *per* and *tim* exhibited significant sex differences [*P*_Treatment×Line×Sex_ = 0.0064 (FDR = 0.0482) and < 0.0001 (FDR = 0.0017), respectively]. Thus, transcriptional abundance differs in canonical clock genes in the DGRP_892 long period line relative to Canton-S B, but these differences are predominantly within genes controlling the negative feedback loop of the clock (i.e., *per* and *tim*), rather than the positive feedback loop (*Pdp1*).

### Candidate gene transcript levels

We assayed expression levels of 26 additional candidate genes predicted by the GWAS as described above. If some of these transcripts are changing in abundance over the 24-h day, that would suggest involvement in the circadian clock. As with the canonical clock genes, we examined reduced ANOVA models for each line separately to determine whether gene expression changed over time, which is one indication that the transcripts are potentially cycling. *Rae1* and *Cpr62Ba* were differentially expressed across time in DGRP_892 in LD (FDR = 0.0433 and 0.0478, respectively), but the differences were confined to one sex only (Table S10). Post-hoc Tukey tests indicated that these genes may be fluctuating in females with a peak at ZT14 and in males with a peak at ZT10-14, respectively (Table S10). JTK_CYCLE analysis indicated that *Rae1* may be rhythmic in females (*P* = 0.0269) but did not find rhythmicity in *Cpr62Ba* in males.

**Fig. 4:**
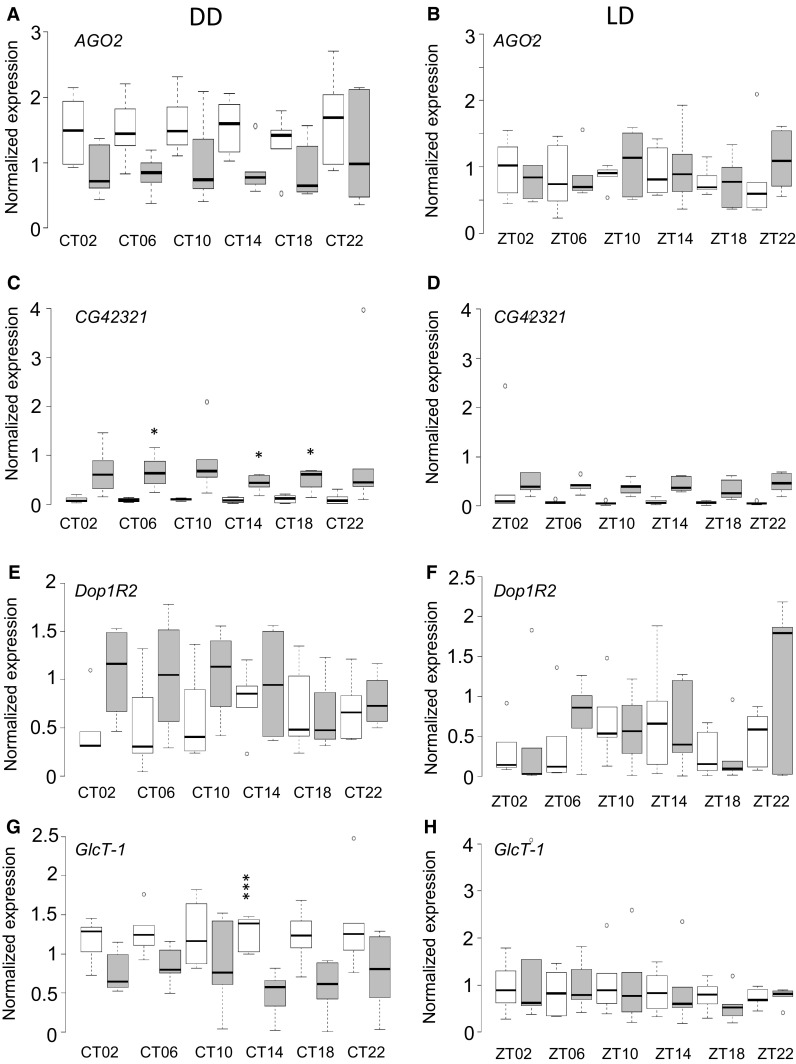
Candidate gene expression profiles of Canton-S B and DGRP_892. Normalized transcript levels are plotted against time as in Fig. 3. **a, b***AGO2*. **c, d***CG42321*. **e, f***Dop1R2*. **g, h***GlcT-1*. The remaining genes are plotted in Figure S6

We were particularly interested in those genes with differential gene expression between Canton-S B and DGRP_892 (Table S11 and S12). Nine genes had differences in gene expression between Canton-S B and DGRP_892 across sexes, times, and lighting conditions: *AGO2* (FDR = 0.0705; marginally significant, but see below), *CG42321* (FDR < 0.0001), *Dop1R2* (FDR = 0.0442), *GlcT-1* (FDR = 0.0450), *GluRIIA* (FDR = 0.0141), *Mdr65* (FDR = 0.0263), *Rae1* (FDR = 0.0206), *Tep4* (FDR = 0.0029), and *tnc* (FDR = 0.0198) (Fig. 4a–h; Fig. S6). Three genes had significant differences in expression between Canton-S B and DGRP_892 that were specific to lighting conditions: *AGO2* (FDR = 0.0016), *GlcT-1* (FDR = 0.0055), and *tnc* (FDR = 0.0353) (Table S11). The expression of *CG42321* tended to be higher in DGRP_892 than Canton-S B under all conditions; CT06, CT14, and CT18 were significantly different in DD (FDR = 0.0269, 0.0193, and 0.0374, respectively) (Table S13). When gene expression for *Dop1R2* and *GluRIIA* was averaged across all timepoints, there were significant differences in gene expression between Canton-S B and DGRP_892. For *Dop1R2*, DGRP_892 had higher average expression (Fig. 4e, f), while for *GluRIIA*, Canton-S B had higher average expression (Fig. S6A and B). Canton-S B had higher *GlcT-1* expression than DGRP_892 at CT14 in DD (*P*_Time_ = 0.0003; FDR = 0.0348) (Fig. 4g, h). *tnc* transcript levels were higher in Canton-S B at all timepoints in LD. The remaining genes showed more complex patterns of expression across time. These line-specific differences in gene expression potentially contribute to the long period in DGRP_892.

Thus, nine candidate genes from the GWAS had differential expression between Canton-S B and DGRP_892, seven of which were predicted to have epistatic effects with other candidate genes or the canonical circadian clock genes (Table S9). Whether these genes are causal in the long period of DGRP_892 remains to be elucidated, but differences in gene expression indicate that they may have a role.

### Behavioral tests of candidate genes with gene expression differences

We tested candidate genes with differences in gene expression for their role in circadian period and rhythmicity index using available mutations and RNAi knockdowns. We tested *CG42321, Cpr62Ba*, and *tnc* using *Minos* {*ET1*} element lines (Bellen et al. 2011).The *Minos* lines have an control line with an identical genetic background, which makes them ideal for candidate gene tests. However, the *Cpr62Ba* and *tnc* stocks had been maintained via crosses to a different strain, which altered the genetic background. Therefore, for these two mutants, we tested flies that were heterozygous as well as homozygous for the *Minos* element. *Minos* allele *CG42321*^MB05800^ had a significant effect on χ^2^ period (Table 1 and Table S15). *tnc*^MB04464^ mutants had significantly altered rhythmicity index (Table 1).

**Table 1:** Differences in circadian phenotypes from control

Gene	Allele	Change in χ^2^ period (h)	Change in RI	Construct type
*CG42321*	MB05800	**− 0.3697**	0.0117	Minos ET1
*Cpr62Ba*	MB12091	0.0408	**−** 0.0101	Minos ET1
*Cpr62Ba* heterozygote	MB12091	**0.3890**	**−** 0.0440	Minos ET1
*tnc*	MB04464	0.0013	**− 0.0762**	Minos ET1
*tnc* heterozygote	MB04464	**0.2877**	**−** 0.0508	Minos ET1
*AGO2*	321	0.2111	0.0223	Deletion
*CG42321*	MI06777	**−** 0.0963	**−** 0.1030	MIMIC
*CG42321* heterozygote	MI06777	**−** 0.1301	**− 0.1463**	MIMIC
*CG42321*	MI08838	**− 1.1054**	**−** 0.0340	MIMIC
*Cpr62Ba*	MI03734	**− 0.7054**	**−** 0.0555	MIMIC
*Cpr62Ba* heterozygote	MI03734	**−** 0.5270	**−** 0.0405	MIMIC
*Cpr62Ba*	MI12486	**− 0.6312**	**−** 0.0569	MIMIC
*Dop1R2*	MI08664	**−** 0.0756	**−** 0.0843	MIMIC
*GlcT-1*	G5974	**− 0.7315**	**−** 0.0439	EP
*GlcT-1* heterozygote	G5974	**− 0.4749**	**− 0.1120**	EP
*GlcT-1*	MI06082	**−** 0.1935	**−** 0.0375	MIMIC
*GluRIIA*	AD9	0.3397	**−** 0.0425	Null
*Mdr65*	KG08723	0.0019	**−** 0.0588	P
*Tep4*	MI13472	**− 0.5409**	**− 0.1329**	MIMIC
*Tep4* heterozygote	MI13472	**− 0.7067**	**−** 0.0628	MIMIC
*Tep4*	EY04656	**− 1.2226**	0.0509	EPgy
*tnc*	EY16369	**−** 0.4067	**−** 0.0174	EPgy
*AGO2*	HMC03828	**0.6828**	**−** 0.0336	TRiP RNAi
*Dop1R2*	JF02043	**−** 0.0744	**−** 0.0248	TRiP RNAi
*Dop1R2*	HMC06293	**−** 0.4868	**0.0445**	TRiP RNAi
*GlcT-1*	HMC06408	**0.9155**	**−** 0.1050	TRiP RNAi
*GluRIIA*	JF02647	**−** 0.4564	**− 0.0418**	TRiP RNAi
*Mdr65*	JF03079	**−** 0.1019	0.0183	TRiP RNAi
*Mdr65*	HMS01449	**−** 0.6712	0.0077	TRiP RNAi
*Rae1*	HMJ21842	0.2046	0.0081	TRiP RNAi
*Tep4*	HMC06319	**0.4025**	**−** 0.0334	TRiP RNAi
*tnc*	HMC05051	**0.8658**	**−** 0.0735	TRiP RNAi
*bru1*	MB05908	0.0876	**− 0.0838**	Minos ET1
*CG11073*	MB07687	**−** 0.0266	**−** 0.0438	Minos ET1
*CG13243*	MB09929	0.0018	**−** 0.0496	Minos ET1
*CG17839*	MB08271	0.1560	**−** 0.0148	Minos ET1
*CG32052*	MB02409	**− 3.5153**	0.0278	Minos ET1
*CG34355*	MB03916	**−** 0.0531	**−** 0.0431	Minos ET1
*CG42672*	MB05883	**−** 0.0018	**−** 0.0357	Minos ET1
*CG6123*	MB02356	**− 1.6312**	**−** 0.0113	Minos ET1
*flw*	MB01707	0.2584	**−** 0.0342	Minos ET1
*Mp*	MB08228	0.0561	**−** 0.0556	Minos ET1
*Prosap*	MB03234	0.0363	**− 0.1054**	Minos ET1
*Ptp99A*	MB04947	0.0328	**−** 0.0278	Minos ET1
*sano*	MB03560	0.0643	0.0022	Minos ET1
*Sh*	MB00560	0.1888	**−** 0.0294	Minos ET1
*SKIP*	MB04854	**−** 0.0250	**− 0.0594**	Minos ET1

The table shows the mean difference in circadian phenotype from control for each mutant/RNAi knockdown tested. A negative difference indicates that the mutant/RNAi knockdown was higher than the control, while a positive difference indicates that the mutant/RNAi knockdown was lower. Significant differences from controls (*P* < 0.05) are indicated in bold. Table S14 lists genotypes and control lines tested. Control lines used for each allele and control line phenotypes can be found in Tables S15, S16, and S17

Furthermore, we tested several genes having *Minos* {*y*[+mDint2]=*MIC*} insertions available in *CG42321, Cpr62Ba, Dop1R2, GlcT-1, Mdr65, Tep4*, and *tnc*. (Venken et al. 2011). In addition, we tested available deletions in *AGO2* and *GluRIIA*, a *P*{*w*[+mC]=*EP*1} insertion in *GlcT-1, P*{*w*[+mC] *y*[+mDint2]=*EPgy2*} insertions in *Tep4* and *tnc*, and a *P*{*y*[+mDint2] *w*[BR.E.BR]=*SUPor-P*} insertion in *Mdr65*. One mutant allele of *CG42321, CG42321*^MI08838^, affected χ^2^ period, but a second allele, *CG42321*^MI06777^, did not (Table 2). Additionally, the *CG42321*^MI06777^ allele affected RI, but only as a heterozygote (Table 1). Both of the *Cpr62Ba*^MI12486^ and *Cpr62Ba*^MI03734^ alleles tested affected χ^2^ when homozygous. The *Cpr62Ba*^M12486^ allele only targets the first intron of the -RB isoform of *Cpr62Ba*, while the *Cpr62Ba*^MI03734^ and *Cpr62Ba*^MB12091^ alleles putatively affect both isoforms (Table S14). The *Dop1R2*^MI08664^ allele affected RI in males only, with a difference of – 0.1297 from the control; no other effects of this mutation were observed (Table 1). The *GlcT-1*^MI06082^ allele had no effect on circadian traits; however, the *GlcT-1*^G5974^ allele did, impacting both χ^2^ and RI. Both *GlcT-1* insertions target the transcribed portion of the gene; however, the *GlcT-1*^G5974^ is upstream of the coding region of the gene, while the *GlcT-1*^MI06082^ allele is just downstream of it. Two alleles of *Tep4, Tep4*^MI13472^ and *Tep4*^EY04656^, affected circadian period and the *Tep4*^MI13472^ allele affected RI as well. In addition, male-specific effects on χ^2^ were observed in the *tnc*^EY16369^ allele.

**Table 2:** Candidate genes from this study with human homologs identified for sleep or chronotype

FlyBaseID	Fly gene symbol	Human gene symbol	DIOPT score^a^	Disease/trait	Refs.
FBgn0051678	*CG31678*	*SUCO*	8	Daytime sleep	Spada et al. (2016)
FBgn0029890	*CG4095*	*FH*	8	Daytime sleep	Spada et al. (2016)
FBgn0016694	*Pdp1*	*HLF*	8	Night sleep	Spada et al. (2016)
FBgn0262614	*pyd*	*TJP2*	8	Night sleep	Spada et al. (2016)
FBgn0032886	*CG9328*	*FAM107B*	7	Night sleep	Spada et al. (2016)
FBgn0052423	*shep*	*RBMS3*	7	Night sleep	Spada et al. (2016)
FBgn0004369	*Ptp99A*	*PTPRG*	6	Daytime sleep	Spada et al. (2016)
FBgn0003975	*vg*	*VGLL2*	6	Night sleep	Spada et al. (2016)
FBgn0054056	*CG34056*	*C1GALT1*	5	Daytime sleep	Spada et al. (2016)
FBgn0054056	*CG34056*	*C1GALT1*	5	Night sleep	Spada et al. (2016)
FBgn0052683	*CG32683*	*ARRB1*	4	Obstructive sleep apnea	Cade et al. (2016)
FBgn0085354	*CG34325*	*FHL5*	3	Night sleep	Spada et al. (2016)
FBgn0031573	*CG3407*	*ZNF311*	1	Daytime sleep	Spada et al. (2016)
FBgn0017590	*klg*	*F11R*	1	Night sleep	Spada et al. (2016)
FBgn0003209	*raw*	*RNASEL*	1	Chronotype	Hu et al. (2016), Jones et al. (2016), Lane et al. (2016)

^a^The DIOPT (Drosophila RNAi Screening Center Integrative Ortholog Prediction Tool) Score indicates the number of data bases the homolog was found in, with a maximum score of 9. (Hu et al. 2011)

Finally, we tested *AGO2, Dop1R2, GlcT-1, GluRIIA, Mdr65, Rae1*, Tep4, and *tnc* via RNAi knockdown (Table 1 and S16). These genes are expressed in adult male and female heads (Brown et al. 2014); thus, it is possible that gene expression occurs in clock neural cells. To test the potential effect of these genes on the circadian clock, we drove expression in circadian clock neural cells by crossing these RNAi constructs to Pdf-GAL4 and tim-GAL4. Pdf-GAL4 expression is confined to 4 of 5 small ventral lateral neurons and the large ventral lateral neurons (Renn et al. 1999) while tim-GAL4 is expressed more broadly, targeting the ventral lateral neurons as well as the dorsal neurons (groups 1–3) and the dorsal lateral neurons (Kaneko and Hall 2000). We first tested whether RNAi knockdown using both GAL4 drivers would affect circadian phenotypes. χ^2^ period was affected in *AGO2, GlcT-1, Tep4*, and *tnc* knockdown animals, while RI was affected in *Dop1R2* and *GluRIIA* knockdowns (Table 1). Closer inspection revealed that the effect on χ^2^ period was due to differences between the RNAi knockdowns and the y[1] v[1]; P{y[+t7.7]=CaryP}attP40 control line crossed to tim-GAL4; the χ^2^ period of crosses to Pdf-GAL4 were in the same direction, but not statistically significant. This raised the interesting possibility that the effects of these genes on circadian period require the larger group of clock-related neurons. Alternatively, the tim-GAL4 driver might have been more efficient at reducing gene expression than the Pdf-GAL4 driver.

In summary, tests of mutants and RNAi knockdown flies further implicated our candidate genes as affecting circadian rhythms. The strongest evidence was for *CG42321, Cpr62Ba, GlcT-1*, and *Tep4* affecting χ^2^ period. Multiple constructs of these genes in varied genetic backgrounds affected circadian behavior. Effects on RI were more fleeting, but evidence suggests that *Dop1R2, Tep4*, and *tnc* alter RI.

### Behavioral tests of candidate genes predicted to have large effect sizes

We also conducted tests on candidate genes predicted to have the largest effect sizes on χ^2^ period and rhythmicity index. We tested available *Minos* insertions in *CG11073, CG13243, CG34355, CG42672, Mp, Prosap, Ptp99A, sano*, and *SKIP*, which were predicted to have large effects on χ^2^ period (Table S5); *bru1, CG32052, CG6123, flw*, and *Sh* were predicted to have large effects on rhythmicity index. Although they were predicted to affect rhythmicity index, *CG32052* and *CG6123* had effects on χ^2^ period, with an increase of 3.52 and 1.63 h over the control period, respectively (Table 1 and Table S17). An attempt to excise the *Minos* element from *CG32052* revealed that the long-period phenotype did not map to this gene. *bru1* had a significant effect on rhythmicity index, differing − 0.0831 from the control. *Prosap* and *SKIP* altered rhythmicity index, though they were predicted to affect circadian period. Thus, mutations in four additional candidate genes impacted circadian period and rhythmicity index.

## Discussion

Here we assessed circadian period (Ʈ) and rhythmicity index (RI) in a natural population of flies. The Ʈ estimates calculated by MESA were derived from the highest peak in the power spectral density for each fly, and the low range of Ʈ estimated with MESA did not agree with the low range of χ^2^ period calculations (see Fig. 1). Low periods of ~ 15 h are rare but have been observed in an ultrafast allele of *period* in flies (Konopka et al. 1994). It has been suggested the low period values calculated using MESA may reflect ultradian rhythms (Dowse and Ringo 1987, 1989); alternatively, recent work proposes that high peaks in the power spectral density corresponding to low circadian periods may actually be harmonics of the true circadian period (Lazopulo and Syed 2016). However, both of these methods enabled us to identify candidate genes that could be verified with further testing.

If we considered the more narrowly distributed χ^2^ to represent the true extent of the variation in the DGRP, it would still be far more variable than estimates of circadian period in humans using a forced desynchrony protocol, which have coefficients of variation in the range of 0.54–0.58% (Czeisler et al. 1999). However, chronotype, a related measure, has a heritability of 0.12–0.47 in humans [reviewed in (Kalmbach et al. 2016)] (Toomey et al. 2015; Jones et al. 2016; Lane et al. 2016); bed time, a similar measure, has a heritability of 0.22 (Gottlieb et al. 2007). Furthermore, heritability for circadian period in mice ranges from 0.21 to 0.55 (Hofstetter et al. 1995, 1999). These studies indicate a strong influence of both genetic and environmental factors on circadian period. Similarly, we found relatively low heritability estimates for circadian period in this study of flies, as observed previously (Emery et al. 1995).

Surveys of natural populations of *Drosophila* have identified latitudinal clines (Kyriacou et al. 2008; Rand et al. 2010; Sawyer et al. 1997) in polymorphic variants of canonical clock genes. These variants are associated with other traits that have a clear link to environmental conditions, particularly temperature: diapause, thermal tolerance, and eclosion (Sawyer et al. 1997; Rand et al. 2010; Kyriacou et al. 2008; Pegoraro et al. 2014; Tauber et al. 2007). Thus, polymorphisms influencing circadian period may be maintained as the result of acclimatization (Kyriacou et al. 2008).

In addition to circadian period, RI was highly variable, ranging from 0.05 to 0.55 in the DGRP. Interestingly, seven lines were classified as arrhythmic in one sex: one line had arrhythmic males, while six lines had arrhythmic females. For the most part, the RI in the opposite sex of these lines was not high (≤ 0.17), indicating that both sexes were at best weakly rhythmic. Given the importance of the circadian clock in regulating biological processes, it is puzzling why arrhythmicity would be maintained in nature. It may be that less robust (i.e., more variable) circadian behavior gives these animals an adaptive advantage. Such an advantage has been observed explicitly in cyanobacteria, where arrhythmic strains had higher growth in constant conditions compared to strains with a functional clock (Woelfle et al. 2004). However, strains with functional clocks were more fit in changing circadian conditions than arrhythmic strains (Woelfle et al. 2004; Ouyang et al. 1998). Wildtype flies produced more eggs in constant light conditions, which produce arrhythmic activity patterns; but constant light also reduced lifespan (Sheeba et al. 2000). Thus, the fitness advantage of variable circadian behavior depends heavily on the environmental conditions encountered. Future work will focus on the discovery of trade-offs between these circadian behaviors and other traits as pleiotropic gene action may be the basis for the maintenance of these differences (Carbone et al. 2006).

Our GWAS identified new candidate genes for circadian behavior. Interestingly, no canonical clock genes were identified for Ʈ, while only one polymorphism in *Pdp1* was associated with RI. Only ten of the genes we identified here had been associated with circadian rhythms previously. This is analogous to linkage and genome-wide association studies in chronotype and sleep in humans (Pagani et al. 2016; Jones et al. 2016; Lane et al. 2016; Hu et al. 2016) and in circadian behavior in mice (Shimomura et al. 2001), where few canonical clock genes were implicated. Further, 24.7% of the associated polymorphisms were intergenic, suggesting that circadian rhythms may be modified via transcriptional regulation, if the polymorphisms fall in enhancer or promoter regions (Massouras et al. 2012; Heap et al. 2009). Comparison of this study to a previous study of sleep in the same population showed some overlap between genes associated with both suites of traits, and these comparisons revealed genes largely involved in developmental processes, including neuronal development. Interestingly, a single candidate gene, *raw*, overlaps with a homolog (*RNASEL*) identified in human GWAS studies of chronotype (Hu et al. 2016; Lane et al. 2016; Jones et al. 2016), and 16 additional genes have homologs identified in human GWAS studies of sleep (Spada et al. 2016; Cade et al. 2016) (Table 2). The homology results should be interpreted with caution but suggest a conserved architecture between flies and humans. Thus, some of the genes we identified have been implicated in mammalian studies of related circadian traits and of sleep.

In this study, we identified a line with a very long circadian period, DGRP_892. Both males and females of this line have circadian periods that average 31 h. Not many other observations of long circadian periods in this range in flies have been made. Konopka and Benzer described an allele of *per* that resulted in a 28.5-h circadian period (Konopka and Benzer 1971); Smith et al. (2008) found 33-h periods resulting from a dominant negative allele of the *casein kinase α* subunit; Rothenfluh et al. (2000) identified long-period mutations in *timeless* created via EMS mutagenesis; and Blau and Young noted a 28-h period when *vrille* expression was altered (Blau and Young 1999). We measured gene expression in this line for several candidate genes on the hypothesis that polymorphic variants in and around the candidate gene might alter gene expression (Heap et al. 2009; Massouras et al. 2012). *per* transcript levels are known to be delayed in *per*^*L*^ mutants (Hardin et al. 1990), which have a long circadian period. We did not find the amino acid substitution of *per* reported to cause the long period (Baylies et al. 1987) in DGRP_892. Nor were there any obvious variants private to DGRP_892 that could affect the clock; yet gene expression in *per* was altered in these animals. *Pdp1* also had altered transcriptional oscillations in DGRP_892, though the effects were smaller. Delays in *Pdp1* expression are positively correlated with altered circadian period (Hardin et al. 1990; Muskus et al. 2007; Chen et al. 2013). These observations suggest differential regulation of both the positive and the negative feedback loop of the circadian clock in DGRP_892, though the effect on the negative feedback loop was more pronounced.

We examined gene expression in 26 candidate genes identified in this study as possible mediators of canonical clock gene expression differences in DGRP_892. We selected genes with candidate polymorphisms in coding as well as non-coding regions. We observed altered gene expression in nine candidate genes (*AGO2, CG42321, Dop1R2, GlcT-1, GluRIIA, Mdr65, Rae1, Tep4*, and *tnc*). With the exception of *Rae1* and *Mdr65*, we also observed changes in circadian behavior in mutants and RNAi knockdowns of these genes. We describe the functions of these genes briefly here. A role for *AGO2* in mammalian circadian rhythms has been reported (Lee et al. 2013). *CG42321* is a predicted gene with an unknown function but is a strong candidate for circadian period in this study. Previous work identified *CG42321* as a target of *Clk* (Abruzzi et al. 2011). A Translating Ribosome Affinity Purification study in fly clock cells identified *Cpr62Ba* as cycling (Huang et al. 2013). *Dop1R2* has a role in neuronal inhibition of sleep-promoting neurons in the dorsal fan-shaped body (Pimentel et al. 2016); here we found that *Dop1R2* affected rhythmicity index. *GlcT-1* has a role in developmental apoptosis (Kohyama-Koganeya et al. 2004), and is predicted to be cycling in mammals (Hughes et al. 2010). Four of the candidate genes—*GluRIIA, Mdr65, Rae1* and *Tep4*—have mammalian homologs which have been reported to be cycling in the SCN and other specific mammalian organs (Hughes et al. 2010). *GluRIIA* encodes a glutamate receptor capable of calcium ion transfer (Han et al. 2015) predicted to cycle in peripheral mouse organs (Hughes et al. 2010). *Mdr65* is a gene involved in the transport of xenobiotic substances across the fly’s neural protective barrier (Mayer et al. 2009). *Rae1* functions to limit synaptic growth (Tian et al. 2011). In addition to cycling in mammals, *Tep4* is a *Drosophila* immune response gene that has been previously shown to be regulated in a circadian manner (Ceriani et al. 2002). Though a direct role of *tenectin (tnc)* in circadian rhythms is unknown in *Drosophila*, its mammalian homolog *RTN3* (*reticulon 3*) is reported to cycle in the mouse liver (Hughes et al. 2010) and SCN (Panda et al. 2002). *tnc* expression is regulated by ecdysone and it encodes an integrin ligand involved in wing morphogenesis; it is also expressed in *Drosophila* CNS (Fraichard et al. 2006, 2010).

Nine genes had differential gene expression in DGRP_892 relative to Canton-S B, and several of these (*GlcT-1, Mdr65*, and *Tep4*; see Table S9) were predicted to interact with canonical circadian clock genes. Yet few of the genes we tested had significant differences in transcript abundance in a 24-h period. *Rae1* was the only gene with significant differences in gene expression at different times. One potential reason for this is that we only assayed six timepoints over the circadian day; additional timepoints may help resolve these differences. In addition, more replication would improve these measures, as the statistical power for measurements of gene expression under different environmental conditions is low relative to differences detectable by genotype (Lin et al. 2016b). Alternatively, transcriptional cycling of molecules may not be required to modify circadian behavior (Ray and Reddy 2016); the genotype-specific differences in expression would then be relevant to the phenotypic differences we observed. Finally, studies have reported that synonymous SNPs may lead to altered protein translation kinetics and thereby altered protein conformation rather than affecting transcription (Kimchi-Sarfaty et al. 2007; Komar et al. 1999). It would be worth exploring whether these unique synonymous variants affect protein functions which in turn result in aberrant circadian phenotypes in DGRP_892.

We also tested *Minos* mutants in 14 genes predicted to have large effect sizes on χ^2^ period and rhythmicity index. *CG6123*, a gene with unknown function, were predicted to affect rhythmicity index but instead increased period. Likewise, *Prosap*, which affects the number of synaptic boutons in the neuro-muscular junction (Harris et al. 2016) and *SKIP*, a potassium-channel interacting protein with a role in olfaction (Tunstall et al. 2012), affected rhythmicity index; however, they were predicted to have effects on period. Only *bru1* affected rhythmicity index as predicted. These pleiotropic mutational effects have been previously observed for sleep as well (Wu et al. 2018; Harbison et al. 2013). This may indicate that polymorphic variants have strong context-dependent effects on sleep and circadian rhythms (Chandler et al. 2017). Alternatively, an improvement in phenotypic modeling may yield more accurate predictions.

In this study, we have identified candidate polymorphisms associated with circadian behavior. Although we have not yet established that these variants are causal, our future goal is to elucidate mechanisms leading to phenotypic variance. This work is part of a systems genetics approach (Ray and Reddy 2016) and has identified previously unappreciated polymorphisms for circadian behavior.

## Materials and methods

### Quantitative circadian rhythm phenotypes

We measured circadian phenotypes on 167 lines of the *Drosophila* Genetic Reference Panel (DGRP), a collection of inbred lines derived from wild-caught flies (Mackay et al. 2012; Huang et al. 2014). Flies were maintained on standard culture medium (http://flystocks.bio.indiana.edu/Fly_Work/media-recipes/bloomfood) at 25 °C and 60–65% relative humidity, under a 12-h light: dark (LD) cycle until circadian measurements were made. We recorded fourteen continuous days of activity in constant darkness (DD) using the *Drosophila* Activity Monitoring System (Trikinetics, Waltham, MA). Activity counts were sampled in 1-min intervals. Flies were placed on 5% sucrose, 1.5% agar food for the duration of the circadian measurements as standard culture medium becomes too dry for the flies to eat after 14 days. The DGRP lines were randomly divided into four equal blocks. We replicated circadian measurements three times for each block of lines. Replicate measurements were conducted at 2-week intervals. Eight flies of each sex per line were measured in each replicate, resulting in circadian measurements for 24 flies per sex per line, or 48 flies per line. We assumed that we would be able to detect differences of 1 h (for MESA and χ^2^ period) and 0.1 for RI at 80% statistical power (in practice, we were able to detect differences in period as low as 0.765 and 0.359 h for MESA and χ^2^ period, and differences as low as 0.045 for RI at 80% power) (Sokal and Rohlf 1995). Eight male and eight female *w*^1118^; *Canton-S B* flies were measured in each replicate as a control. Although the effects of social exposure on circadian rhythms are not known, both social exposure and mating status affect sleep in flies (Ganguly-Fitzgerald et al. 2006; Isaac et al. 2010); we therefore collected virgin males and females from each line and housed them at 30 flies per same-sex vial for 4 days prior to making the activity measurements. We discarded the measures from flies that did not live through the recording period; we also discarded the first day of recording as the flies were recovering from CO_2_ exposure. All other measurements, including any outliers, were retained in the analysis.

We calculated the rhythmicity index (RI) and period length (Ʈ) for the DGRP population. To make these calculations, we first summed the activity counts into 30-min bins, which reduces the potential for spurious patterns due solely to the choice of bin size (Dowse and Ringo 1994). To determine RI, we calculated the autocorrelelogram for each fly using the equation

$${r_k}=\frac{{\mathop \sum \limits_{{t=1}}^{{N - k}} ({x_t} - \bar {x})({x_{t+k}} - \bar {x})}}{{\mathop \sum \limits_{{t=1}}^{N} {{({x_t} - \overline {x} )}^2}}}$$

where ${r_k}$ is the autocorrelation coefficient at lag $k$, ${x_t}$ is the activity level at time $t$, ${x_{t+k}}$ is the activity level at time $t+k$, and $\overline {x}$ is the mean activity level for the fly (Chatfield 2003). We defined RI as the correlation coefficient ${r_k}$ of the third highest peak in the autocorrelelogram (Levine et al. 2002). In addition to a sinusoidal component, the autocorrelelograms of some flies exhibit a linear trend, which can skew the rhythmicity index (Levine et al. 2002). To account for this linear trend, we first fitted a regression line to each autocorrelelogram. If the autocorrelelogram had a nominally significant linear component (*P* < 0.05), we adjusted the RI for that trend by subtracting the calculated RI of the predicted regression line. We classified flies as rhythmic or arrhythmic using the 95% confidence interval $\frac{2}{{\sqrt N }}$, where $N$ is the number of activity measurements (Levine et al. 2002; Chatfield 2003). Since we summed our activity measures into 30-min bins, *N* = 672 and the 95% confidence interval for the autocorrelation coefficient was ${r_k}$ = 0.0772. Flies with an RI below this value were classified as arrhythmic.

We used two methods to calculate Ʈ: Maximum Entropy Spectral Analysis (MESA) (Burg 1967), and the chi-squared periodogram (Enright 1965). MESA analysis assumes that the time series data is composed of an autoregressive function $X\left( t \right)$ and a stochastic or noise function ${Z_t}$ (Levine et al. 2002; Burg 1967). At a given time $t$, $X(t)$ is

$$X\left( t \right)=~{a_1}{X_{t - 1}}+~{a_2}{X_{t - 2}}+ \cdots {a_n}{X_{t - n}}+~{Z_t}$$

where the $a$ coefficients are estimated from the data, $n$ is number of terms, $t$ is time, and $Z$ is the stochastic function. A frequency-based spectrum $S(\omega )$ can then be calculated via Fourier transform from the equation

$$S\left( \omega \right)=~\frac{P}{{{{\left| {1 - \mathop \sum \nolimits_{{k=1}}^{p} {a_{k~}}{e^{ - i\omega k}}} \right|}^2}}}$$

where $P$ is the output power or spectral density, $p$ is the number of coefficients, and $i=\sqrt { - 1}$ (Levine et al. 2002; Burg 1967; Dowse 2007, 2009; Dowse and Ringo 1989). Circadian period can then be estimated as the time at which the maximum spectral density peak occurs (Dowse and Ringo 1989). We used BATCHMES, a freely available program, to implement MESA [H. Dowse, personal communication, and (Dowse 2007)].

Furthermore, power spectral density graphs were examined for each rhythmic fly; for most flies, the spectral density contained a single dominant peak. In the graphs for some flies, we did not observe an interpretable peak. These calculations for these flies were removed from further analysis.

We also calculated period using the chi-squared periodogram. This method first assumes a period $P$ for the data. If the activity data are broken up into units of period $P$, then the differences between these units can be used to calculate a χ^2^ statistic to determine how well the assumed period fits the actual data. We used an in-house C^#^ program (R. Sean Barnes) to estimate the ${Q_p}$ statistic using the equation

$${Q_p}=\frac{{KN\mathop \sum \nolimits_{{h=1}}^{P} {{\left( {{M_h} - M} \right)}^2}}}{{\mathop \sum \nolimits_{{i=1}}^{N} {{\left( {{X_i} - M} \right)}^2}}}$$

where ${X_i}~$ is the activity counts for a given unit of time in a data set of $N$ values, $M$ is the mean of all $N$ values, and ${M_h}$ is the mean activity counts for each time unit for $K$ values (Refinetti 2004; Enright 1965). We calculated ${Q_p}$ for a range of possible periods$~P$, from 12 h to 32 h (Refinetti 2004; Enright 1965). ${Q_p}$ has a χ^2^ probability distribution with $P - 1$ degrees of freedom (Refinetti 2004). If ${Q_p}~$was not significant according to the χ^2^ distribution, we did not estimate the period; if it was, we used the highest ${Q_p}$ value to estimate circadian period (Refinetti 2004). If we judged the fly to be arrhythmic using the rhythmicity index criteria, we did not estimate circadian period.

### Quantitative genetic analyses

We partitioned the variance in each circadian parameter using the ANOVA model: *Y* = *µ* + *B* + *S* + *L*(*B*) + *S* × *L*(*B*) + *R*(*B*) + *S* × *R*(*B*) + *R* × *L*(*B*) + *S* × *R* × *L*(*B*) + *ε*, where *L* (line), *B* (block) and *R* (replicate) are random effects, *S* (sex) is a fixed effect, and *ε* is the error variance. We used reduced models to partition the variance for each sex separately. We estimated variance components using the restricted maximum likelihood (REML) method. We calculated broad sense heritability as *H*^2^ = (*σ*^2^_*L*_ + *σ*^2^_*SL*_)/(*σ*^2^_*L*_ + *σ*^2^_*SL*_ + *σ*^2^_*E*_) for sexes combined, where *σ*^2^_*L*_ is the variance component among lines, *σ*^2^_*SL*_ is the line-by-sex variance component, and *σ*^2^_*E*_ is the sum of all other sources of variation. We used *H*^2^ = *σ*^2^_*L*_/(*σ*^2^_*L*_ + *σ*^2^_*E*_) to estimate broad-sense heritability for sexes separately. We calculated the cross-sex genetic correlation *r*_*MF*_ as *σ*^*2*^_*L*_/√(*σ*^2^_*LM*_ × *σ*^2^_*LF*_), where *σ*^2^_*L*_ is the variance component among lines for sexes combined, *σ*^2^_*LM*_ is the variance component among lines for males separately, and *σ*^2^_*LF*_ is the variance component among lines for females separately. We calculated the genetic correlation *r*_*G*_ between circadian and sleep traits as *r*_*G*_ = *cov*_12_/√(*σ*_*L1*_^2^ × *σ*_*L2*_^2^) (Falconer and Mackay 1996), where *cov*_12_ is the covariance between traits 1 and 2, and *σ*_*L1*_^2^ and *σ*_*L2*_^2^ are the among-line variances for traits 1 and 2, respectively.

### Genotype-phenotype associations

We associated the line mean of each circadian rhythm parameter with all 1,920,276 segregating sites in the DGRP having a minor allele frequency of 0.05 or greater using the DGRP2 web-based analysis (http://dgrp2.gnets.ncsu.edu) (Huang et al. 2014), which implements the FaST-LMM algorithm (Lippert et al. 2011). This analysis first adjusts the phenotypic line means for the effects of *Wolbachia pipientis* infection and major chromosomal inversions [*In(2L)t, In(2R)NS, In(3R)P, In(3R)K*, and *In(3R)Mo*] where present. The DGRP2 web-based analysis fits a linear mixed model that incorporates any cryptic genetic relatedness present in the lines. The linear mixed model is $y={\mathbf{X}}b+{\mathbf{Z}}u+e$, where $y$ is the phenotypic line mean adjusted for effects of *Wolbachia* infection and segregating polymorphic inversions, ${\mathbf{X}}$ represents the covariance relationship matrix for the fixed variant effect $b$, ${\mathbf{Z}}$ is the incidence matrix for the random polygenic effect $u$, and $e$ is the residual effect. False discovery rate (FDR) corrections for multiple tests were computed using the method of Benjamini and Hochberg (Benjamini and Hochberg 1995). As line DGRP_892 had a very long period, we removed DGRP_892 from the GWA of circadian period, as well as the epistatic analysis of circadian period described below since leaving it in would violate assumptions of normality (Mackay et al. 2012). The power to detect significant associations varies with normalized effect size (*a*/*σ*_G_) and minor allele frequency. We expected to have the power to detect normalized effect sizes of 0.5 or greater where minor alleles were common, i.e., the frequency was close to 0.5 (Mackay et al. 2009). We used DIOPT to identify human homologs of candidate genes identified in the genome-wide analysis (Hu et al. 2011).

### Pairwise epistatic associations

All possible variant pairs having minor allele frequencies of 0.15 or greater were tested for epistatic interactions. Pairs were pruned for high linkage disequilibrium (*r*^2^ ≥ 0.8) in 100-bp windows shifted in 10-bp increments, leaving 666,202 variants for the analysis. We applied the model *Y* = *µ* + *M1* + *M2* + *M1* × *M2* + *ε*, where *M1* is the first marker considered, *M2* is the second marker considered, and ε is the error term. Phenotypes were adjusted for the presence of *Wolbachia* and inversions as well as the top 11 genotypic principal components. We ran the model using FastEpistasis 2.03 (Schupbach et al. 2010), refined the results and obtained *P*-values using the *F* distribution with appropriate degrees of freedom, and retained those interactions (*i.e*., *M1* × *M2* terms) having a *P*-value of 1 × 10^− 7^ or less. These analyses were performed separately for males and females.

### Assessment of selected candidate genes in a long-period line

One of the lines, DGRP_892, had an unusually long circadian period. We characterized gene expression over time in the heads of males and females (Hardin et al. 1990) from this line for 29 candidate genes implicated by the GWA, and under two lighting conditions. We also examined gene expression changes over time in this line for three canonical clock genes: *per, tim*, and *Pdp1. w*^*1118*^;Canton-S B (hereafter referred to as Canton-S B) was used as a control for comparison to DGRP_892 as it had normal circadian behavior (Fig. S7).

### Sample preparation

DGRP_892 and Canton-S B control flies were maintained under standard culture medium, 25 °C, 60–75% relative humidity and lighting conditions (LD) as outlined above. After eclosion, 30 virgin males and 30 virgin females were collected and maintained in sex-separate vials for 3 days. Gene expression was measured in fly heads for two separate lighting conditions: constant darkness (DD), and LD. For the DD condition, flies were first entrained in an incubator in LD for 3 days and then transferred to DD. On the 3rd day of DD, flies were flash-frozen on dry ice every 4 h starting at circadian time (CT) CT02 and stored at -80 °C until further processing. For the LD assay flies were collected and flash-frozen in − 80 °C every 4 h starting at Zeitgeber time (ZT) ZT02 after 6 days in LD. Frozen flies were then transferred to dry ice to remove the heads from the bodies. We replicated this procedure three times to produce 144 samples (2 conditions, LD and DD; 3 biological replicates, 2 sexes, 2 genotypes, and 6 time points).

### RNA extraction

Thirty flies per sample were decapitated on dry ice by gently tapping on the micro centrifuge tubes; heads were transferred to Omni-ruptor tubes (Omni International, Kennesaw, GA) with 4 RNase/DNase-free metal beads (Omni International, Kennesaw, GA) and homogenized in 125 µl of Trizol reagent (Thermo Fisher Scientific, Grand Island, NY) using the Omni-bead Ruptor (Omni International, Kennesaw, GA). The tubes were centrifuged briefly after homogenization and an additional 375 µl of Trizol was added to make the total volume of 500 µl; the mixture was incubated at room temperature for 5 min. Next, 100 µl of chloroform (Mallinckrodt Baker, Center Valley, PA) was added to each tube and mixed for 15 s by shaking. The tubes were incubated at room temperature for 3 min and then centrifuged at 4 °C, 13,000 rpm for 15 min. The aqueous phase was transferred to new Eppendorf tubes and 20 µl of 3M sodium acetate (Quality Biological, Gaithersburg, MD) was added to each sample. Afterward, to eliminate proteins from nucleic acid samples, 250 µl of phenol–chloroform (Sigma, St. Louis, MO) was added and the samples were centrifuged at 4 °C, 13,000 rpm for 15 min. The aqueous phase was transferred to new tubes and 3 µl glycogen (Qiagen, Valencia, CA) and 300 µl of isopropanol (Mallinckrodt Baker, Center Valley, PA) were added followed by centrifugation at 4 °C, 13,000 rpm for 30 min. The supernatants were discarded while avoiding the pellet. The pellets were washed twice in 75% ethanol, centrifuging at 4 °C, 7500 rpm for 5 min. Pellets were air dried for 10–15 min and dissolved in 87.5 µl of RNase-free water (Quality Biological, Gaithersburg, MD). DNase treatment was performed on RNA samples to eliminate any genomic DNA contamination using 10 µl Buffer RDD (Qiagen, Valencia, CA) and 2.5 µl DNase-I stock solution (Qiagen, Valencia, CA) followed by incubation at room temperature for 10 min. The RNA samples were stored at − 80 °C until further processing. RNA was purified using the RNAeasy Kit (Qiagen, Valencia, CA), per the manufacturer’s instructions.

Single-stranded cDNA was made from total RNA using the High-Capacity cDNA Reverse Transcription Kit per the manufacturer’s instructions (Thermo Fisher Scientific, Grand Island, NY). A total of 250 ng RNA was used for each 20 µl reaction. The reverse transcription reactions were performed on a PTC-250 thermocycler (MJ Research, Foster City, CA).

### Real-time quantitative PCR

The cDNA samples were diluted by 20 × for all unknown samples. To create a standard curve, equal portions of each sample were combined and diluted by 5 ×, 20 ×, 100 ×, and 500 ×. PCR reactions were carried out in 384-well plates (Thermo Fisher Scientific, Grand Island, NY) using SYBR Green PCR Master Mix (Thermo Fisher Scientific, Grand Island, NY) according to the manufacturer’s instructions. The PCR reactions were performed on an Applied Biosystems QuantStudio™ 12K Flex Real-Time PCR System using the manufacturer’s protocol (Thermo-Fisher Scientific, Grand Island, NY). All primers were designed using Oligo Analyzer Software 3.1 (Integrated DNA Technologies, Coralville, Iowa). *Actin-5c* was used as an internal control for the total RNA content in each sample. Primer sequence lengths ranged from 19 to 22 bases and were synthesized by IDTDNA (Integrated DNA technologies, Coralville, Iowa). All primer sequences assayed are provided in Table S9.

### Quality control and normalization

Gene expression was estimated from the raw data obtained from QuantStudio™ 12K Flex Real-Time PCR System using standard curves. We first checked the *r*^2^ values of the standard curve by fitting the linear regression of the log transformation of the quantity and the cycle threshold (CT) values. Four different amounts of total RNA (1000, 200, 40 and 8 ng) were used as the known quantities in the standard curve. We found that *r*^2^ value was low for some genes and could be improved after removing the smallest quantity of RNA. To be consistent, therefore, we used the highest three values for quantity in the standard curve for all genes. None of the samples were estimated to have quantities below 40 ng.

Each plate had two technical replicates for each RNA sample under each light/sex/genotype/candidate gene condition. After fitting the standard curve, we averaged the two technical replicates for use in subsequent data analyses. As the samples were necessarily distributed across several plates, we used Quantile (Q) normalization (Bolstad et al. 2003) to correct the bias caused by potential plate effects (Mar et al. 2009). In addition, we also normalized the data using the quantity of *Actin-5c* in each genotype/sex/rep/condition as a reference (Ling and Salvaterra 2011; Ponton et al. 2011) by dividing the Q-normalized quantities of the target genes by the Q-normalized quantities of *Actin-5c*, which did not significantly vary over time or genotype (Fig. S8). The resulting ratios were used in the data analysis detailed below.

### Candidate gene expression analysis

We examined the differences in gene expression using the ANOVA model *Y* = *µ* + *L* + *S* + *Tr* + *T* + *L* × *S* + *L* × *Tr* + *L* × *T* + *S* × *Tr* + *S* × *T* + *Tr* × *T* + *L* × *S* × *Tr* + *L* × *S* × *T* + *L* × *Tr* × *T* + *S* × *Tr* × *T* + *L* × *Tr* × *S* × *T* + *ε*, where *Y* is gene expression, *L* and *S* are as defined previously, *Tr* is lighting condition (LD or DD), *T* is time, and *ε* is the error term. We used reduced models to examine gene expression differences in Canton-S B and DGRP_892 by lighting condition, sex, and time. To determine whether the transcripts within a given line and lighting condition might be cycling over the circadian day, we applied the reduced model *Y* = *µ* + *S* + *T* + *S* + *T* × *S* + *ε* and used post-hoc Tukey analysis to determine which time points were significantly different from one another. To control for multiple tests, we calculated false-discovery rates using the method of Benjamini and Hochberg (Benjamini and Hochberg 1995). Genes having significant differential expression over time were then analyzed using JTK_CYCLE (Hughes et al. 2010). JTK_CYCLE applies the Jonckheere-Terpstra-Kendall algorithm to a range of potential circadian period lengths assuming a symmetrical cosine distribution and reports the period of the data, if rhythmic, and a *P*-value (Hughes et al. 2010).

### Verification of candidate genes

We tested circadian behavior in *P*-element mutants and RNAi knockdown flies in 10 candidate genes identified by the GWAS which either had significant differences in gene expression between DGRP_892 and Canton-S B or exhibited evidence of transcriptional cycling. We tested *AGO2, CG42321, Cpr62Ba, Dop1R2, GlcT-1, GluRIIA, Mdr65, Rae1, Tep4*, and *tnc* (Table S14). We tested available *Minos* elements (*Mi{ET1}, Mi{y[*+*mDint2]*=*MIC}*) (Bellen et al. 2011; Venken et al. 2011), *P*-element insertion lines (Bellen et al. 2011, 2004), imprecise excisions (Hain et al. 2010), null alleles (Petersen et al. 1997), and RNAi knockdown lines (Ni et al. 2011; Perkins et al. 2015). Lines with *Mi{ET1}* insertions were created in an isogenic *w*^1118^ background (*w*^1118^[5905]) (Bellen et al. 2011) ; hence we used this background as a control. We used a y[1] w[1] [1495] control with Mi{y[+mDint2]=MIC} and all other *P*-element insertions. We used *w*^1118^ [3605] as a control for the *AGO2* and *GluRIIA* mutants; these deletions were not viable as homozygotes and were tested as heterozygotes. The TRiP RNAi constructs were derived from two lines: y[1] v[1]; P{y[+t7.7]=CaryP}attP40 and y[1] v[1]; P{y[+t7.7]=CaryP}attP2. If the RNAi transgene was inserted on the 2nd chromosome attP40 site, we used y[1] v[1]; P{y[+t7.7]=CaryP}attP40 (36304) as a control. Likewise, if the RNAi transgene was inserted on the 3rd chromosome attP2 site, we used P{y[+t7.7]=CaryP}attP2 (36303) as a control (Perkins et al. 2015). All RNAi lines and their respective controls were crossed with Pdf-GAL4 and tim-GAL4 driver lines (Kaneko and Hall 2000). The Pdf-GAL4 line drives gene expression in the ventrolateral neurons of the brain (Renn et al. 1999). The tim-GAL4 driver drives tim expression in the ventrolateral neurons as well as additional neuronal subsets (DN1, DN2, and DN3 neurons as well as dorsolateral neurons) (Kaneko and Hall 2000). Note that the cross between tim-GAL4 and UAS-Rae1-RNAi was lethal.

We also tested available *Minos* elements *Mi{ET1}* for candidate genes having large predicted combined-sex effect sizes for χ^2^ period and RI: *bru1, CG11073, CG13243, CG17839, CG32052, CG34355, CG42672, CG6123, flw, Mp, Prosap, Ptp99A, sano, Sh*, and *SKIP* (Table S14). The *w*^1118^[5905] line was used as the control for all mutations. Note that the *sano* insertion was tested as a heterozygote balanced over *CyO* as homozygous mutants were lethal.

Flies were maintained under standard culture (cornmeal-molasses-agar medium, 25 °C, 60–75% relative humidity) and lighting conditions (12-h light: dark cycle) (LD). Five virgin male and five females were used for setting up cultures in an LD incubator. For RNAi knockdowns, five male flies containing UAS-constructs were crossed with five GAL4 females. Twenty virgin males and twenty virgin females were collected and maintained in sex-separate vials for 3 days at LD. Individual flies were then transferred to DAM2 monitors (Trikinetics, Waltham, MA) for for 4 days in LD followed by 7 days in DD to assess the entrainment and free-running behavior. Flies were set up on 5% sucrose, 2% agar food. Sixteen flies per sex per line were tested, for a total of 32 flies per line. We then calculated χ^2^ and RI as indicated above. For all *Minos* element, *P*-element, and deletion mutants, we analyzed the data using the ANOVA model *Y* = *µ* + *G* + *S* + *G* × *S* + *ε*, where *G* is genotype and *S* is sex. For the RNAi knockdown flies, we used the model *Y* = *µ* + *GAL4* + *UAS* + *S* + *GAL4* × *UAS* + *GAL4* × *S* + *UAS* × *S* + *GAL4* × *UAS* × *S* + *ε*, where *GAL4* is the effect of the *Pdf* or *tim GAL4* driver, *UAS* is the effect of the RNAi knockdown line, and *S* is sex. Mutant and RNAi knockdowns were compared to controls in a pairwise fashion using a post-hoc Tukey test.

## Electronic supplementary material

Below is the link to the electronic supplementary material.

Supplementary material 1 (PPT 1206 KB)

Supplementary material 2 (DOCX 20 KB)

Supplementary material 3 (XLSX 1118 KB)
