# Supplementary material for: Genome-Wide Association Study of Circadian Behavior in Drosophila melanogaster
Source: Behav Genet. 2018 Oct 19;49(1):60–82. doi: 10.1007/s10519-018-9932-0 (PMC6326971; doi:10.1007/s10519-018-9932-0)
Supplement: Supplementary file 1 — Supplementary material 1 (PPT 1206 KB) [file 10519_2018_9932_MOESM1_ESM.ppt]

## Slide 1
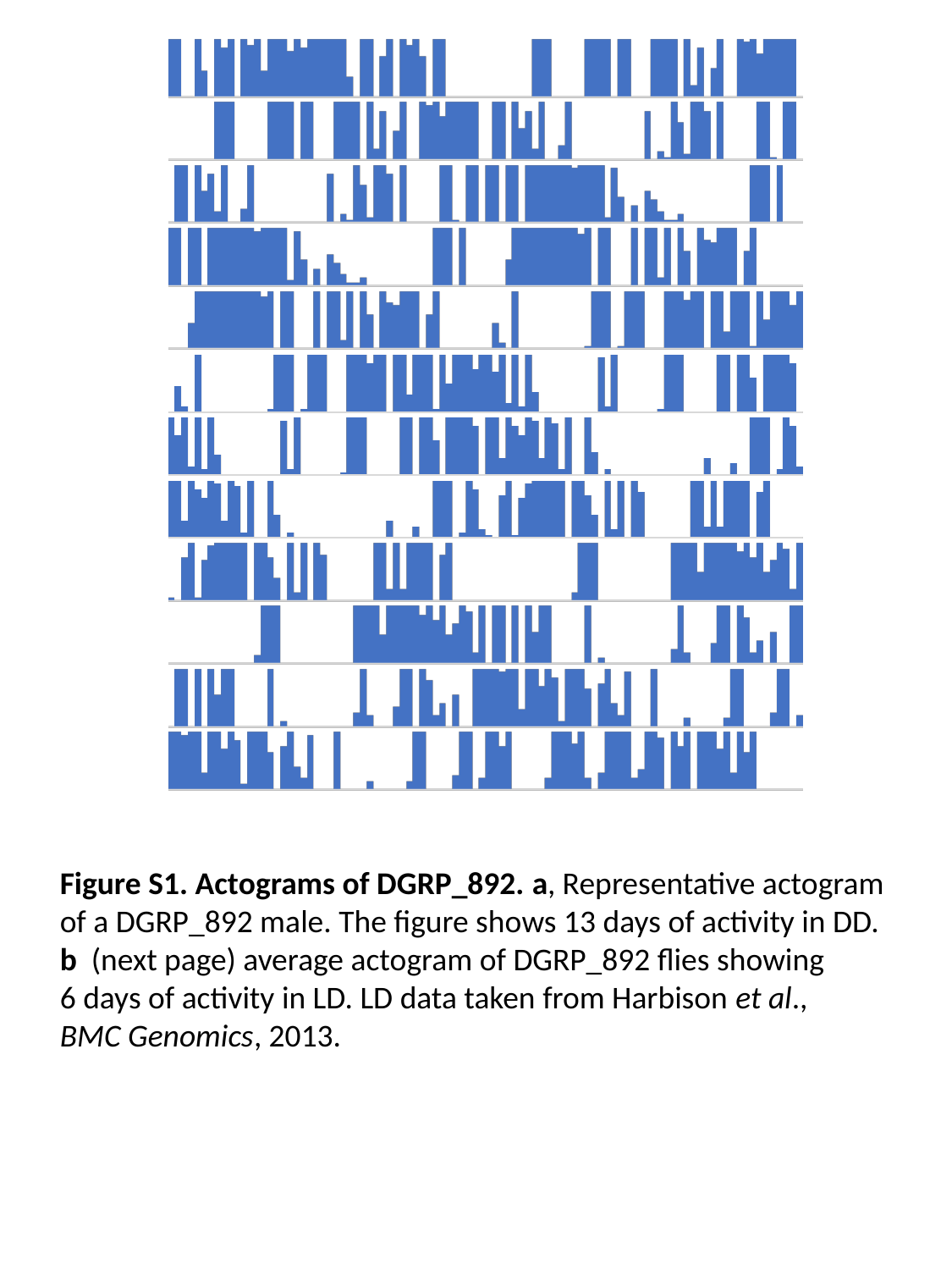

Figure S1. Actograms of DGRP_892. a, Representative actogram
of a DGRP_892 male. The figure shows 13 days of activity in DD.
b (next page) average actogram of DGRP_892 flies showing
6 days of activity in LD. LD data taken from Harbison et al.,
BMC Genomics, 2013.

## Slide 2
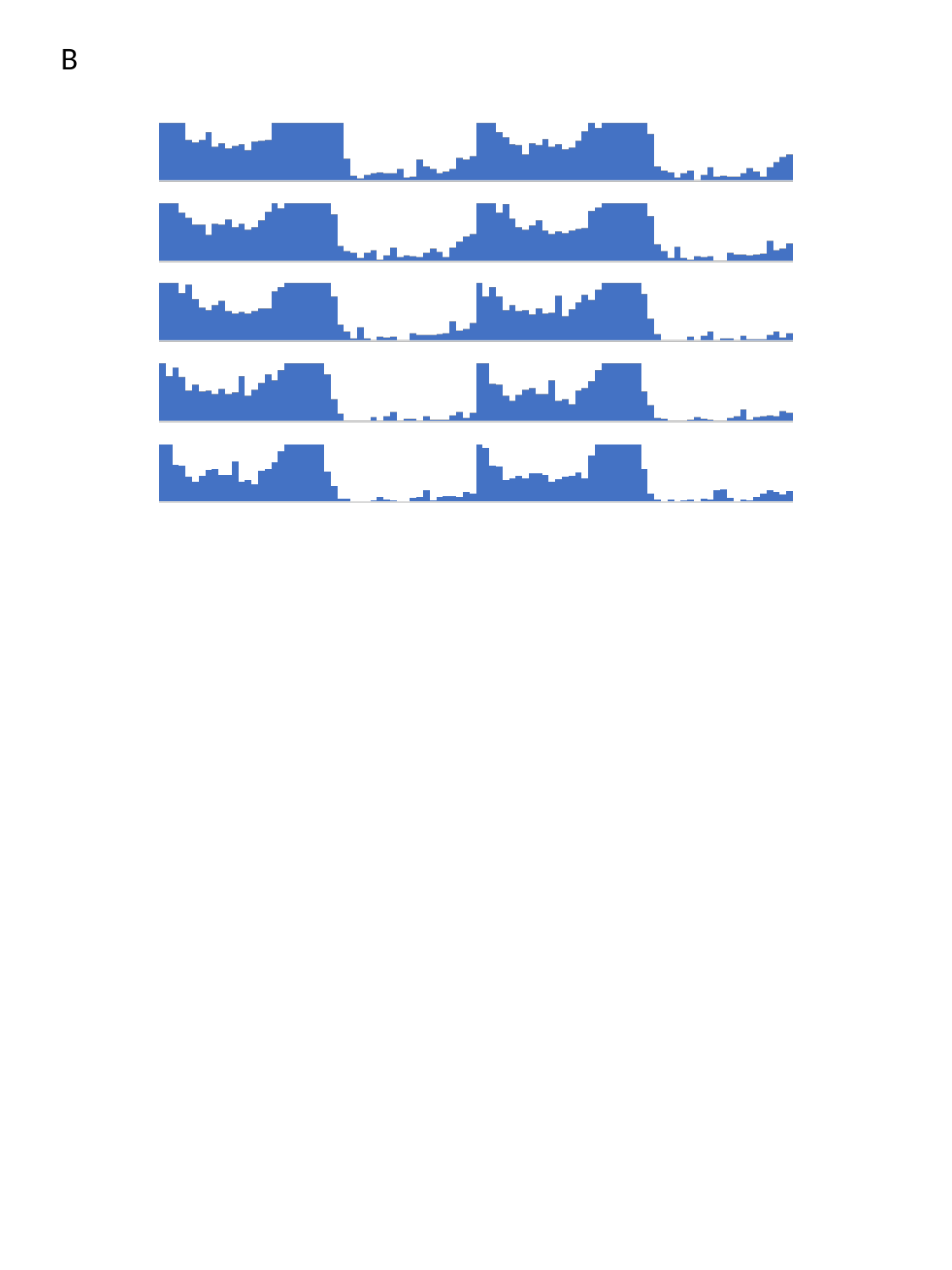

B

## Slide 3
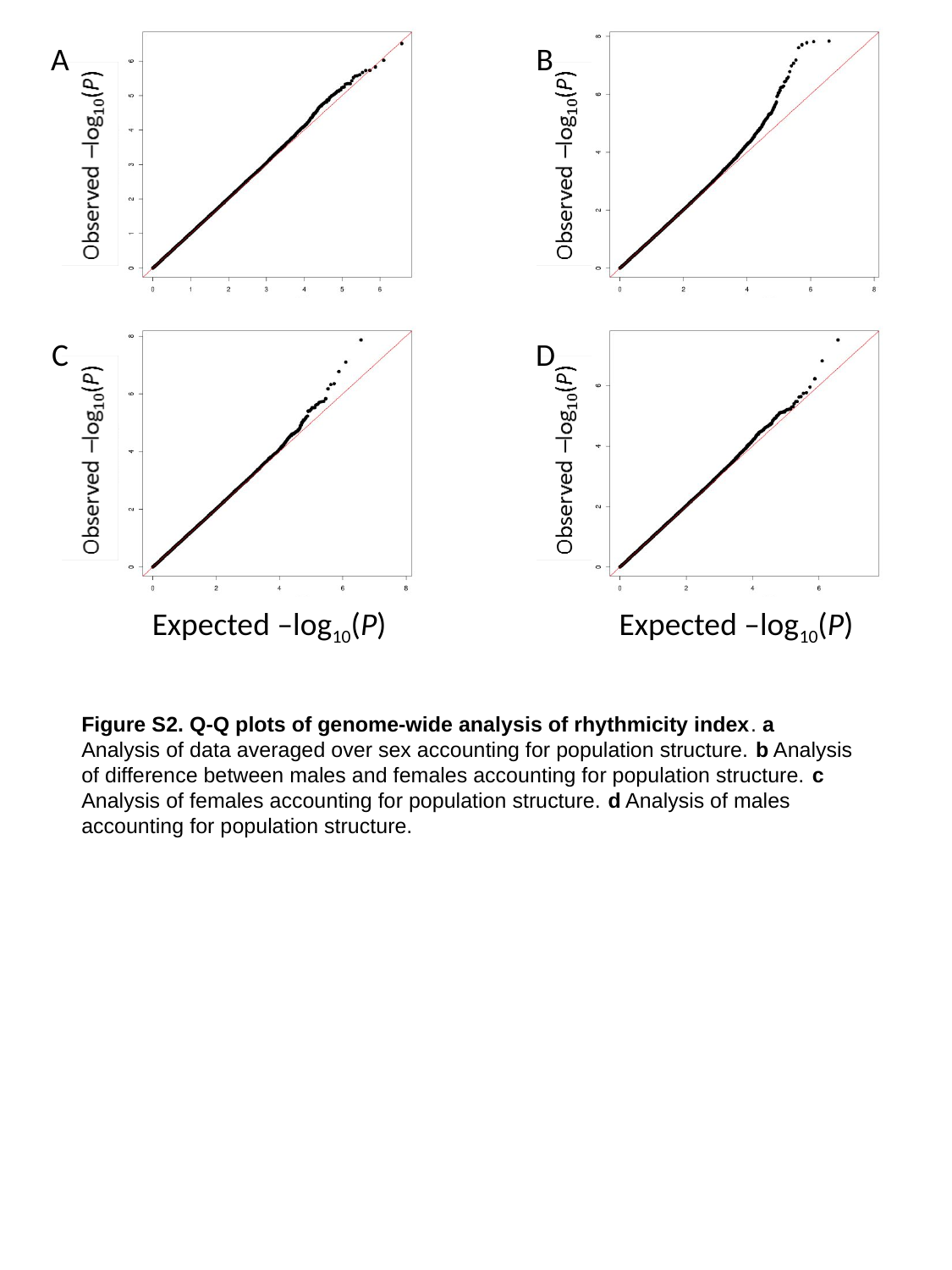

A
B
C
D
Expected –log10(P)
Expected –log10(P)
Figure S2. Q-Q plots of genome-wide analysis of rhythmicity index. a Analysis of data averaged over sex accounting for population structure. b Analysis of difference between males and females accounting for population structure. c Analysis of females accounting for population structure. d Analysis of males accounting for population structure.

## Slide 4
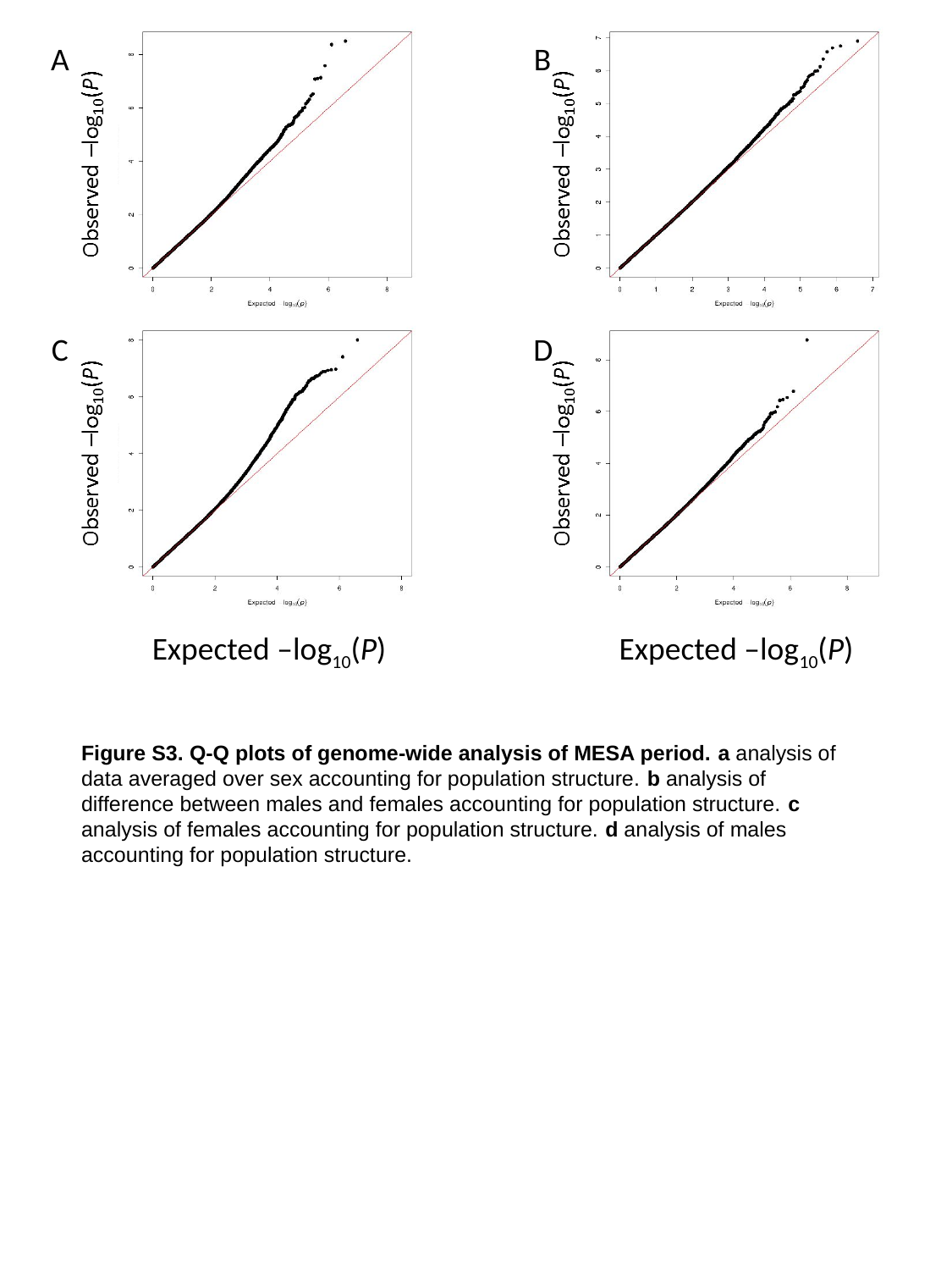

A
B
C
D
Expected –log10(P)
Expected –log10(P)
Figure S3. Q-Q plots of genome-wide analysis of MESA period. a analysis of data averaged over sex accounting for population structure. b analysis of difference between males and females accounting for population structure. c analysis of females accounting for population structure. d analysis of males accounting for population structure.

## Slide 5
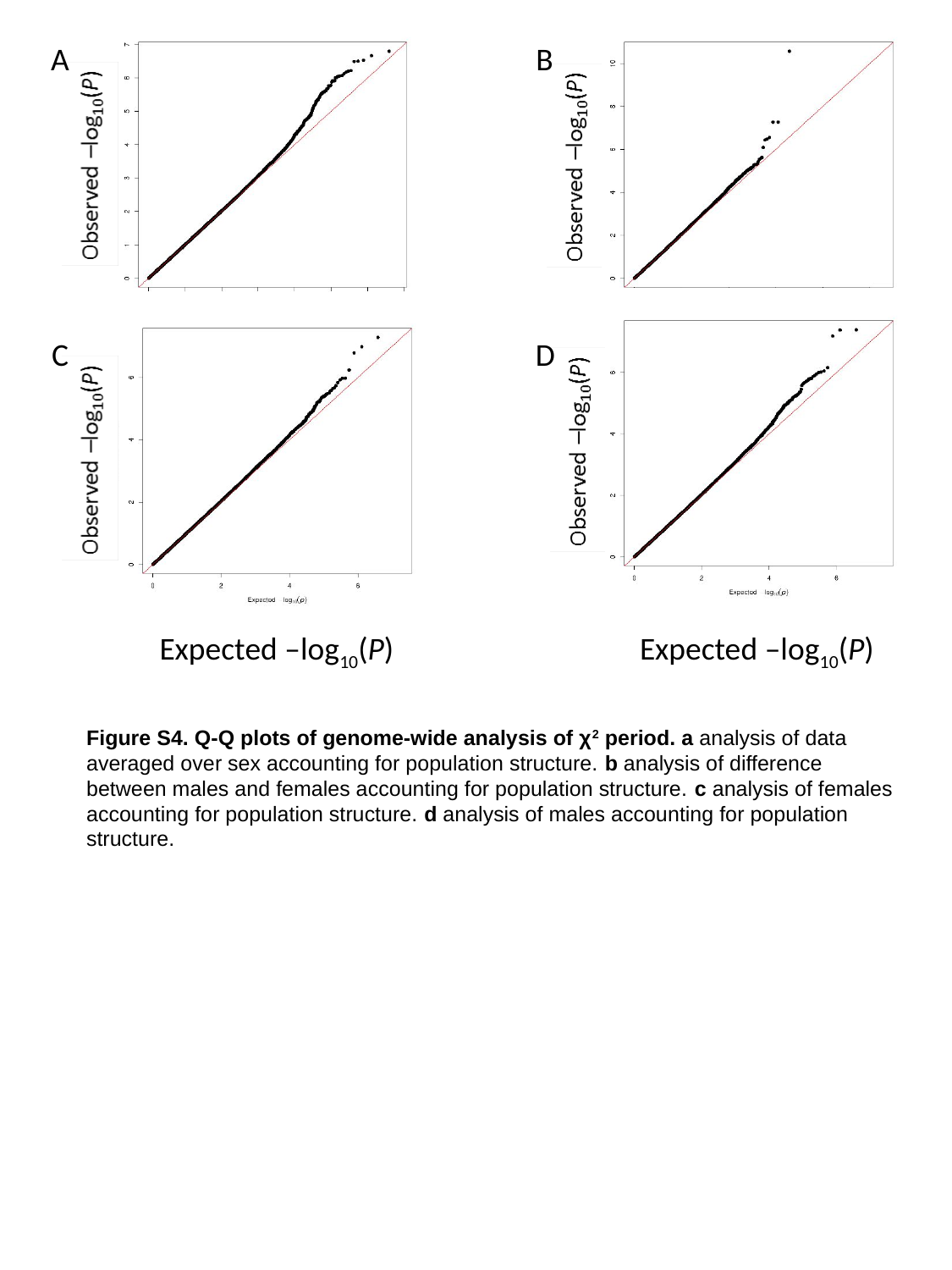

A
B
C
D
Expected –log10(P)
Expected –log10(P)
Figure S4. Q-Q plots of genome-wide analysis of χ2 period. a analysis of data averaged over sex accounting for population structure. b analysis of difference between males and females accounting for population structure. c analysis of females accounting for population structure. d analysis of males accounting for population structure.

## Slide 6
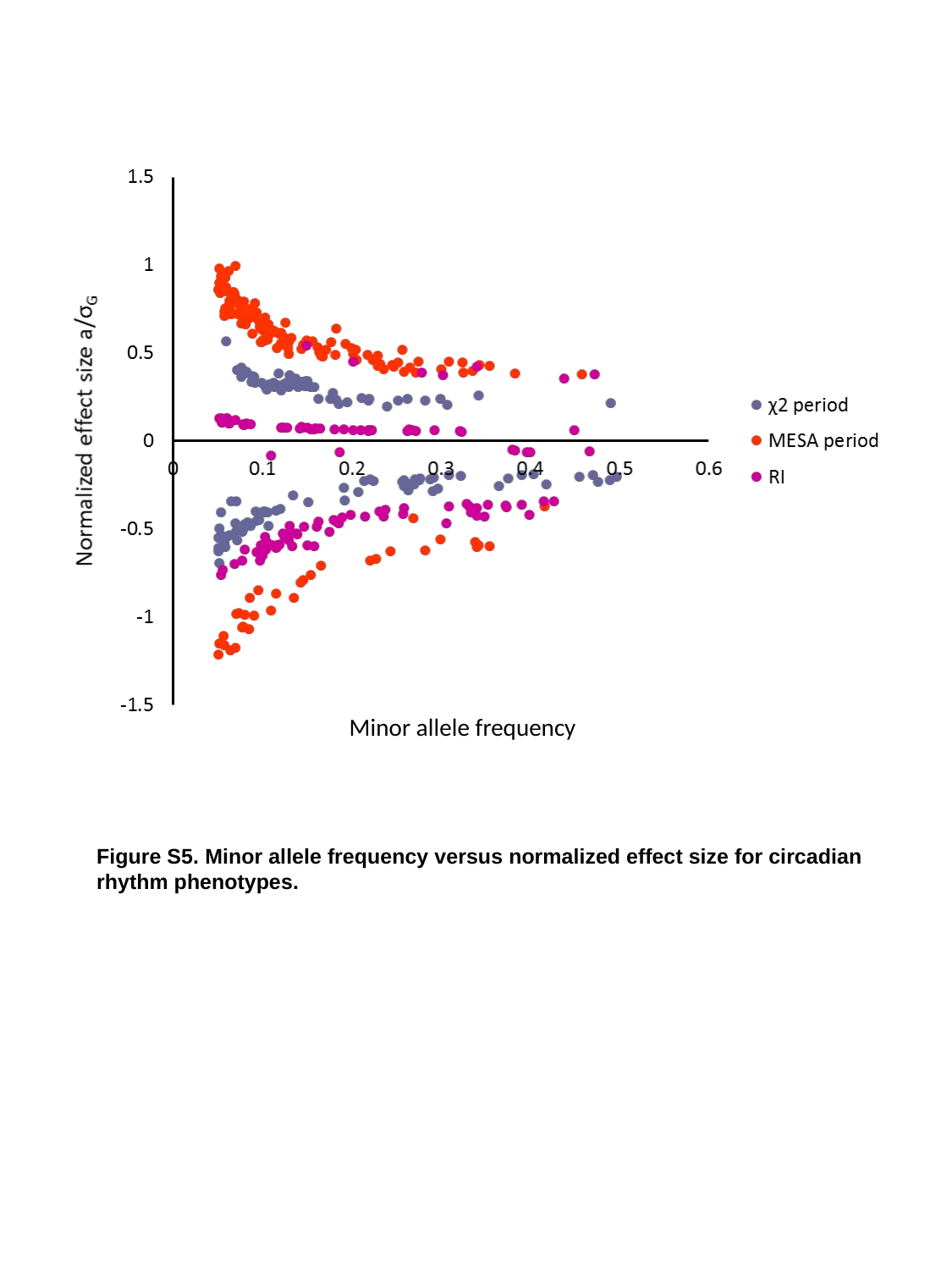

Minor allele frequency
Figure S5. Minor allele frequency versus normalized effect size for circadian rhythm phenotypes.

## Slide 7
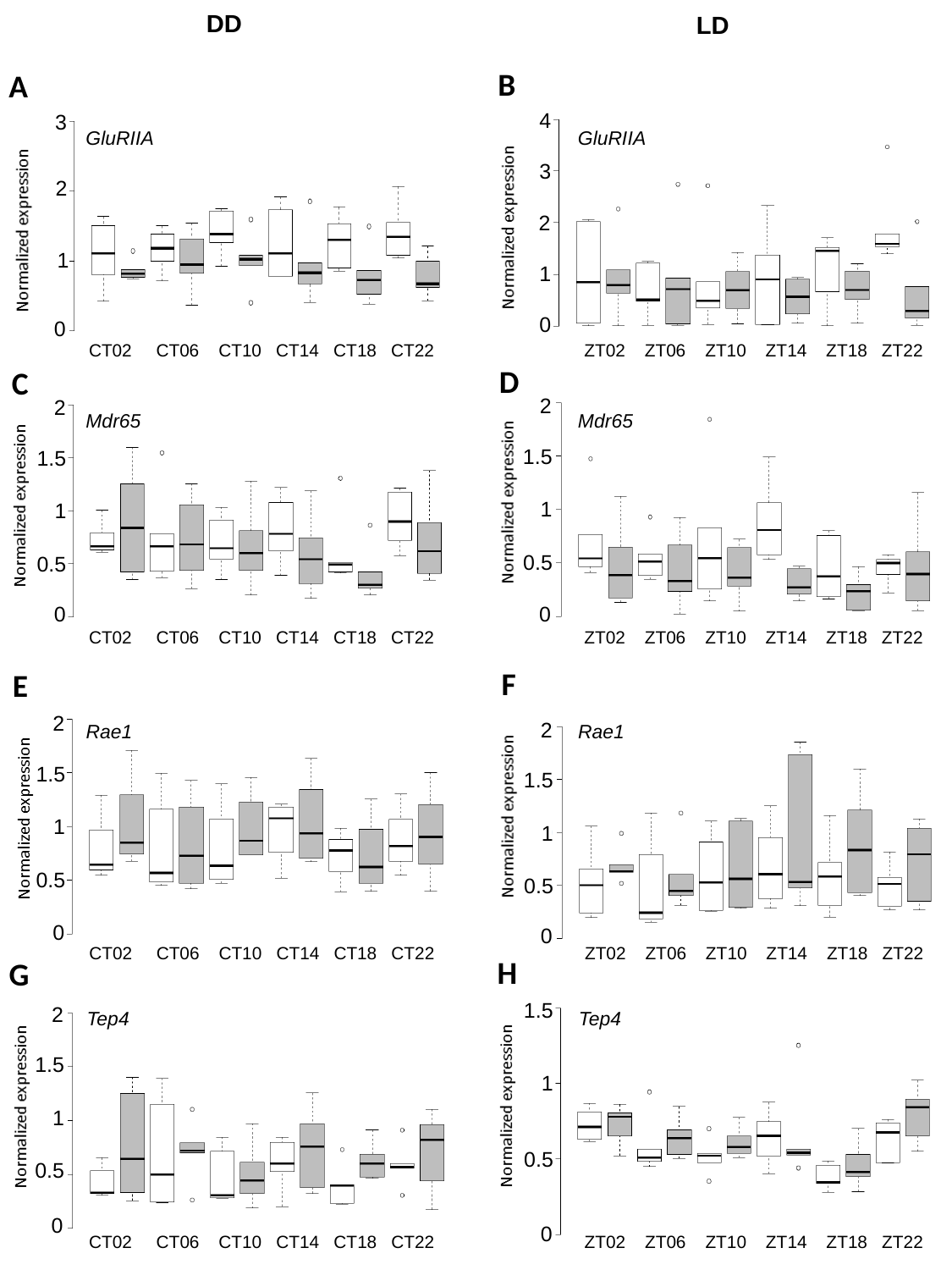

DD
LD
B
A
4
3
GluRIIA
GluRIIA
3
2
2
1
1
0
0
 CT02 CT06 CT10 CT14 CT18 CT22
 ZT02 ZT06 ZT10 ZT14 ZT18 ZT22
D
C
2
2
Mdr65
Mdr65
1.5
1.5
1
1
0.5
0.5
0
0
 CT02 CT06 CT10 CT14 CT18 CT22
 ZT02 ZT06 ZT10 ZT14 ZT18 ZT22
F
E
2
2
Rae1
Rae1
1.5
1.5
1
1
0.5
0.5
0
0
 CT02 CT06 CT10 CT14 CT18 CT22
 ZT02 ZT06 ZT10 ZT14 ZT18 ZT22
H
G
1.5
2
Tep4
Tep4
1.5
1
1
0.5
0.5
0
0
 CT02 CT06 CT10 CT14 CT18 CT22
 ZT02 ZT06 ZT10 ZT14 ZT18 ZT22

## Slide 8
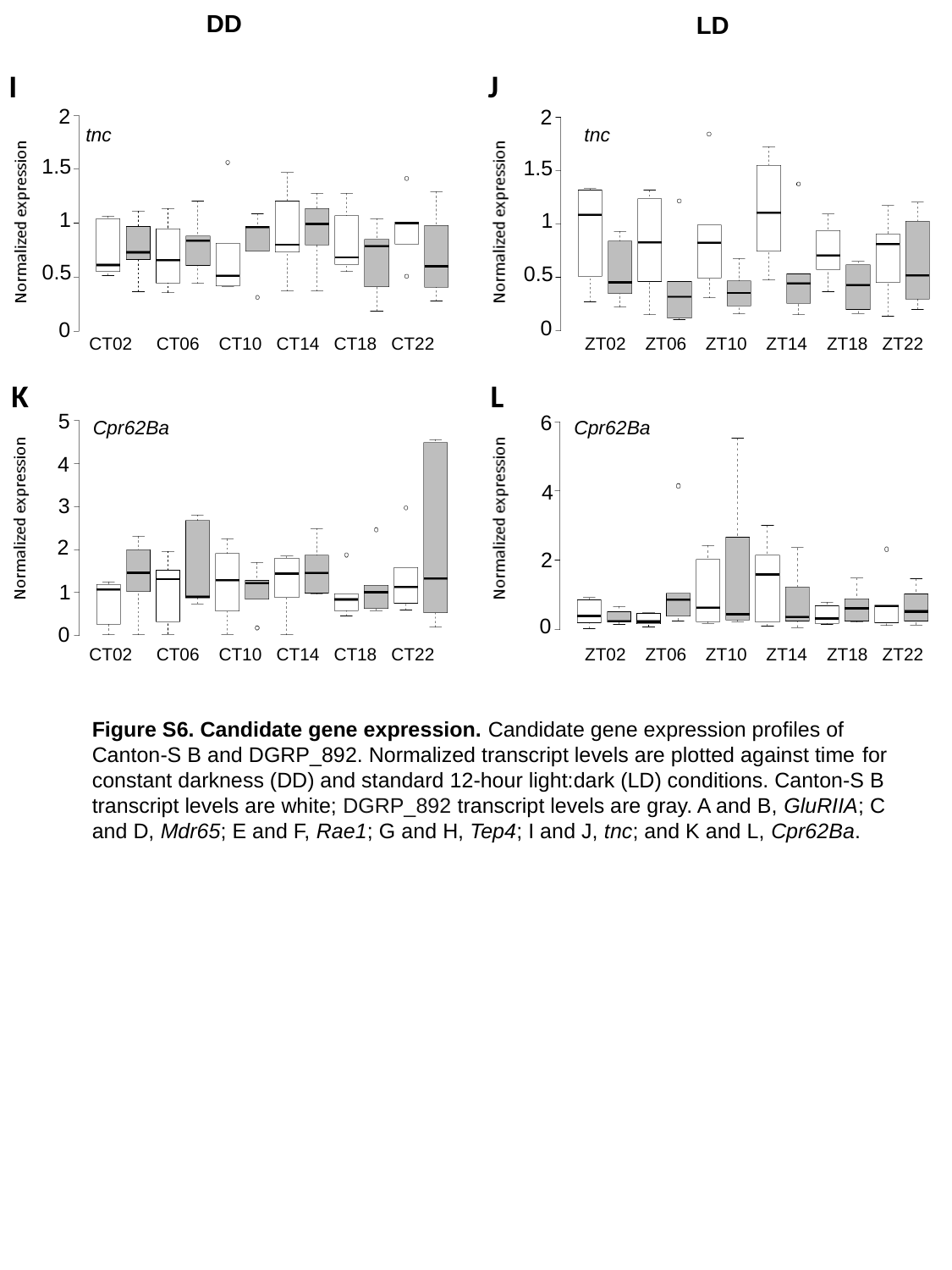

DD
LD
I
J
2
2
tnc
tnc
1.5
1.5
1
1
0.5
0.5
0
0
 CT02 CT06 CT10 CT14 CT18 CT22
 ZT02 ZT06 ZT10 ZT14 ZT18 ZT22
K
L
5
6
Cpr62Ba
Cpr62Ba
4
4
3
2
2
1
0
0
 CT02 CT06 CT10 CT14 CT18 CT22
 ZT02 ZT06 ZT10 ZT14 ZT18 ZT22
Figure S6. Candidate gene expression. Candidate gene expression profiles of Canton-S B and DGRP_892. Normalized transcript levels are plotted against time for constant darkness (DD) and standard 12-hour light:dark (LD) conditions. Canton-S B transcript levels are white; DGRP_892 transcript levels are gray. A and B, GluRIIA; C and D, Mdr65; E and F, Rae1; G and H, Tep4; I and J, tnc; and K and L, Cpr62Ba.

## Slide 9
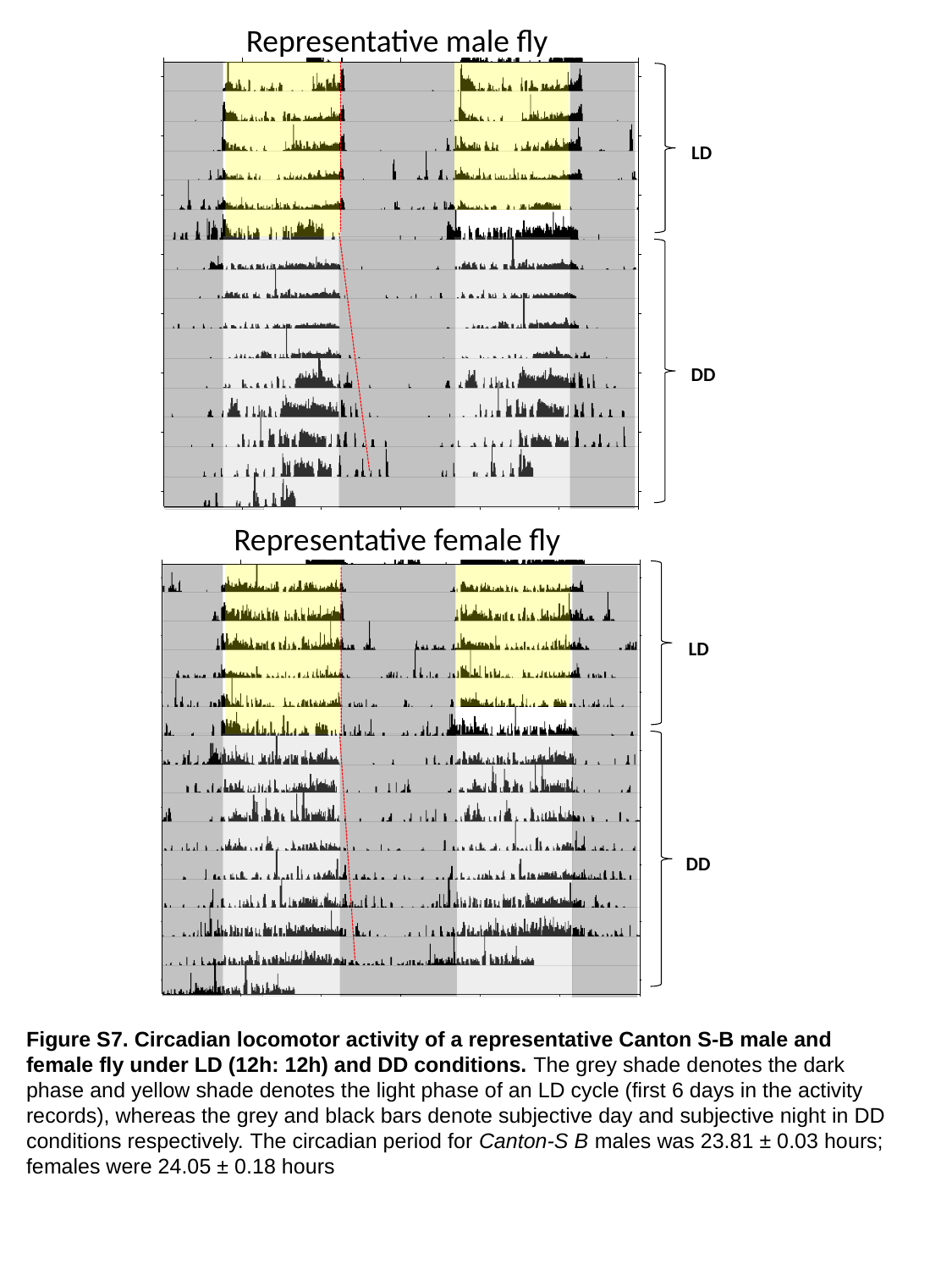

Representative male fly
LD
DD
Representative female fly
LD
DD
Figure S7. Circadian locomotor activity of a representative Canton S-B male and female fly under LD (12h: 12h) and DD conditions. The grey shade denotes the dark phase and yellow shade denotes the light phase of an LD cycle (first 6 days in the activity records), whereas the grey and black bars denote subjective day and subjective night in DD conditions respectively. The circadian period for Canton-S B males was 23.81 ± 0.03 hours;
females were 24.05 ± 0.18 hours

## Slide 10
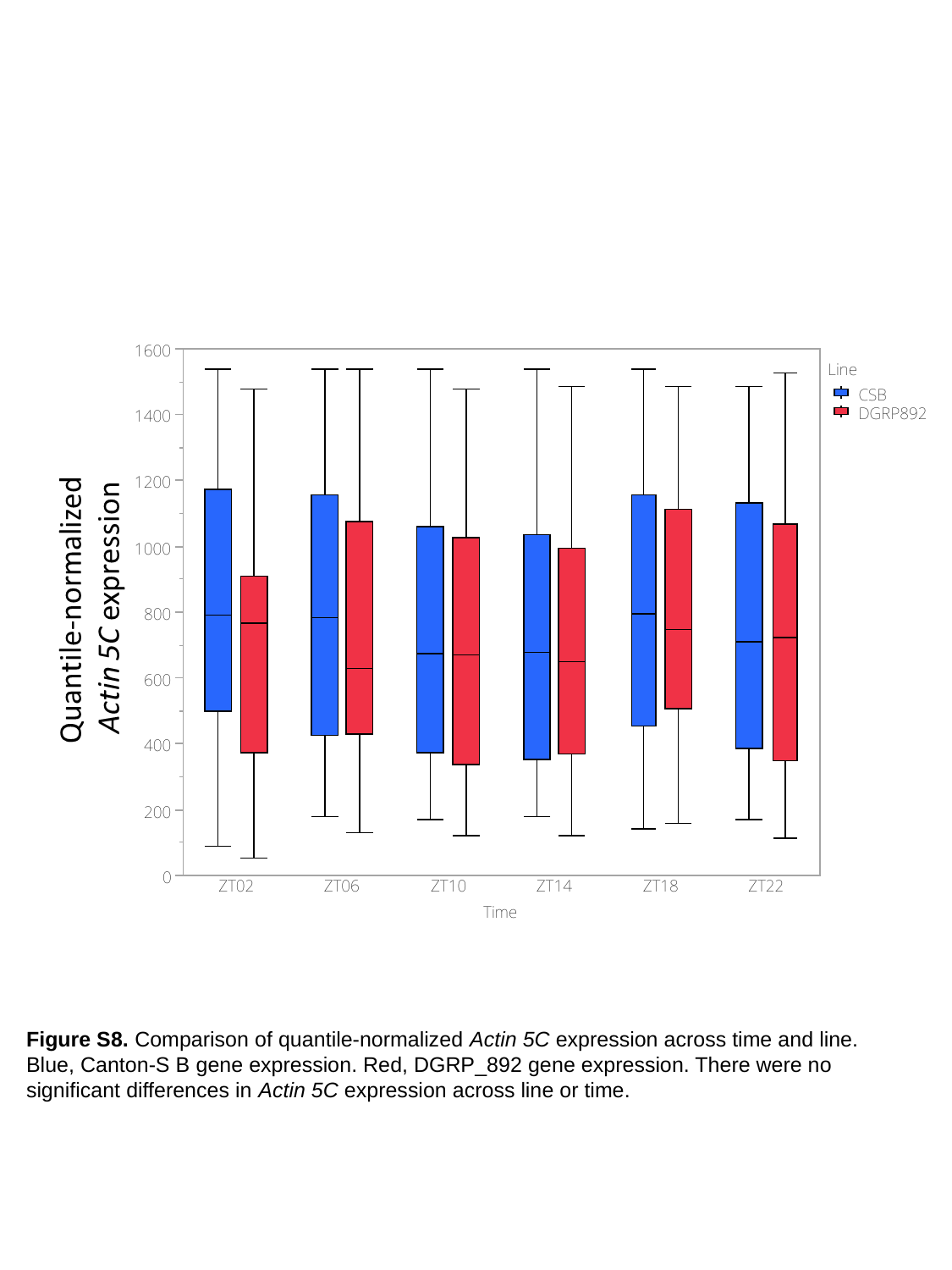

Figure S8. Comparison of quantile-normalized Actin 5C expression across time and line. Blue, Canton-S B gene expression. Red, DGRP_892 gene expression. There were no significant differences in Actin 5C expression across line or time.
