# Supplementary material for: Genome-Wide Association Study of Circadian Behavior in Drosophila melanogaster
Source: Behav Genet. 2018 Oct 19;49(1):60–82. doi: 10.1007/s10519-018-9932-0 (PMC6326971; doi:10.1007/s10519-018-9932-0)
Supplement: Supplementary file 2 — Supplementary material 2 (DOCX 20 KB) [file 10519_2018_9932_MOESM2_ESM.docx]

Table S1. Quantitative genetic analyses of circadian rhythm phenotypes in the DGRP.

| **Phenotype** | | **Analysis** | **Source** | ***d*.*f*.** | **MS^a^** | **F** | ***P*** | ***σ*^2^(×1000)** | ***H*^2^** | ***r*_MF_** |
| --- | --- | --- | --- | --- | --- | --- | --- | --- | --- | --- |
| Rythmicity Index (RI) | Combined Sex | Block | 3 | 423.53 | 0.98 | 0.4073 | 0.00 | 0.43 | 0.62 |  |
|  | |  | Sex | 1 | 1137.58 | 10.62 | 0.0017 | Fixed |  |  |
|  | |  | Line(Block) | 163 | 360.07 | 3.44 | <0.0001 | 6.16 |  |  |
|  | |  | Sex×Line(Block) | 163 | 95.03 | 5.97 | <0.0001 | 3.86 |  |  |
|  | |  | Rep(Block) | 8 | 108.66 | 2.73 | 0.0514 | 0.11 |  |  |
|  | |  | Sex×Rep(Block) | 8 | 30.59 | 1.93 | 0.0548 | 0.06 |  |  |
|  | |  | Rep×Line(Block) | 324 | 25.35 | 1.59 | <0.0001 | 0.65 |  |  |
|  | |  | Sex×Rep×Line(Block) | 320 | 15.95 | 1.36 | <0.0001 | 0.60 |  |  |
|  | |  | Error | 6224 | 11.71 | -- | -- | 11.70 |  |  |
|  | | Males | Block | 3 | 597.49 | 2.49 | 0.0638 | 0.42 | 0.45 |  |
|  | |  | Line(Block) | 163 | 222.36 | 12.01 | <0.0001 | 9.94 |  |  |
|  | |  | Rep(Block) | 8 | 45.12 | 2.48 | 0.0127 | 0.11 |  |  |
|  | |  | Line×Rep(Block | 323 | 18.59 | 1.75 | <0.0001 | 1.14 |  |  |
|  | |  | Error | 3060 | 10.60 | -- | -- | 10.58 |  |  |
|  | | Females | Block | 3 | 31.49 | 0.10 | 0.9577 | 0.00 | 0.41 |  |
|  | |  | Line(Block) | 163 | 237.50 | 10.39 | <0.0001 | 9.88 |  |  |
|  | |  | Rep(Block) | 8 | 95.66 | 4.22 | 0.0001 | 0.19 |  |  |
|  | |  | Line×Rep(Block | 321 | 22.91 | 1.79 | <0.0001 | 1.38 |  |  |
|  | |  | Error | 3164 | 12.78 | -- | -- | 12.78 |  |  |
| MESA Period (hrs.) | | Combined Sex | Block | 3 | 11.19 | 0.11 | 0.9565 | 0.00 | 0.17 | 0.70 |
|  | |  | Sex | 1 | 2997.22 | 110.53 | <0.0001 | Fixed |  |  |
|  | |  | Line(Block) | 163 | 103.15 | 3.01 | <0.0001 | 1798.00 |  |  |
|  | |  | Sex×Line(Block) | 156 | 35.73 | 1.95 | <0.0001 | 1060.48 |  |  |
|  | |  | Rep(Block) | 8 | 28.16 | 2.87 | 0.1126 | 4.89 |  |  |
|  | |  | Sex×Rep(Block) | 8 | 11.03 | 0.61 | 0.7661 | 0.00 |  |  |
|  | |  | Rep×Line(Block) | 323 | 17.06 | 0.93 | 0.7409 | 0.00 |  |  |
|  | |  | Sex×Rep×Line(Block) | 305 | 18.40 | 1.39 | <0.0001 | 624.60 |  |  |
|  | |  | Error | 5284 | 13.21 | -- | -- | 13273.49 |  |  |
|  | | Males | Block | 3 | 32.33 | 0.30 | 0.8249 | 0.00 | 0.19 |  |
|  | |  | Line(Block) | 162 | 105.92 | 4.37 | <0.0001 | 4276.34 |  |  |
|  | |  | Rep(Block) | 8 | 33.77 | 1.43 | 0.1836 | 10.36 |  |  |
|  | |  | Line×Rep(Block | 319 | 24.35 | 1.38 | <0.0001 | 1024.93 |  |  |
|  | |  | Error | 2741 | 17.63 | -- | -- | 17637.16 |  |  |
|  | | Females | Block | 3 | 25.35 | 0.81 | 0.4942 | 0.00 | 0.14 |  |
|  | |  | Line(Block) | 157 | 36.86 | 3.33 | <0.0001 | 1426.06 |  |  |
|  | |  | Rep(Block) | 8 | 8.47 | 0.78 | 0.6170 | 0.00 |  |  |
|  | |  | Line×Rep(Block | 309 | 11.13 | 1.32 | 0.0004 | 155.11 |  |  |
|  | |  | Error | 2543 | 8.44 | -- | -- | 8611.72 |  |  |
| χ^2^ Period (hrs.) | | Combined Sex | Block | 3 | 25.66 | 1.25 | 0.3010 | 4.57 | 0.39 | 0.92 |
|  | |  | Sex | 1 | 38.45 | 29.09 | 0.0001 | Fixed |  |  |
|  | |  | Line(Block) | 163 | 16.41 | 8.11 | <0.0001 | 395.34 |  |  |
|  | |  | Sex×Line(Block) | 156 | 1.31 | 1.60 | 0.0002 | 30.55 |  |  |
|  | |  | Rep(Block) | 8 | 7.12 | 4.67 | 0.0017 | 12.72 |  |  |
|  | |  | Sex×Rep(Block) | 8 | 0.87 | 1.08 | 0.3739 | 0.00 |  |  |
|  | |  | Rep×Line(Block) | 323 | 1.55 | 1.90 | <0.0001 | 63.07 |  |  |
|  | |  | Sex×Rep×Line(Block) | 304 | 0.82 | 1.45 | <0.0001 | 20.20 |  |  |
|  | |  | Error | 5314 | 0.56 | -- | -- | 571.22 |  |  |
|  | | Males | Block | 3 | 15.83 | 1.33 | 0.2729 | 4.72 | 0.39 |  |
|  | |  | Line(Block) | 162 | 9.97 | 7.83 | <0.0001 | 500.71 |  |  |
|  | |  | Rep(Block) | 8 | 3.97 | 3.24 | 0.0014 | 9.90 |  |  |
|  | |  | Line×Rep(Block | 319 | 1.28 | 1.92 | <0.0001 | 84.46 |  |  |
|  | |  | Error | 2759 | 0.67 | -- | -- | 679.55 |  |  |
|  | | Females | Block | 3 | 11.09 | 1.04 | 0.3850 | 3.55 | 0.40 |  |
|  | |  | Line(Block) | 157 | 8.08 | 8.13 | <0.0001 | 369.62 |  |  |
|  | |  | Rep(Block) | 8 | 4.36 | 4.63 | <0.0001 | 14.84 |  |  |
|  | |  | Line×Rep(Block | 308 | 1.00 | 2.24 | <0.0001 | 90.72 |  |  |
|  | |  | Error | 2555 | 0.45 | -- | -- | 449.93 |  |  |

*d*.*f*, degrees of freedom; MS, Type III Mean Squares; F, F ratio statistic; *P*, *p*-value for F ratio statistic; σ^2^, variance component; *H*^2^, broad sense heritability; *r*_MF_, cross-sex genetic correlation. ^a^ MS for rhythmicity index multiplied by 10^3^.

**Table S3.** Combined-sex phenotypic and genetic correlations among circadian traits. Phenotypic correlations are above the diagonal; genetic correlations are below the diagonal. Bold indicates that the correlation is significantly different from zero.

| Trait | MESA period | χ^2^ period | RI |
| --- | --- | --- | --- |
| MESA period |  | **0.5311**^a^ | 0.0531 |
| χ^2^ period | **0.7083**^a^ |  | -0.1249 |
| RI | **0.1780** | **-0.1545** |  |

^a^ Correlations include DGRP_892. Without DGRP_892, the phenotypic and genetic correlations between MESA and χ^2^ period are 0.3884 and 0.6091, respectively.

**Table S4. Phenotypic and genetic correlations with sleep phenotypes.** Bold indicates that the correlation is significantly different from zero at a Bonferroni-corrected *P*-value of 0.0036. A) phenotypic correlations and B) genetic correlations with sleep data collected in a standard 12:12 light:dark cycle; C) phenotypic correlations and D) genetic correlations with sleep data collected in constant darkness.

A)

| Trait | MESA period | χ^2^ period | RI |
| --- | --- | --- | --- |
| Night sleep | 0.1056 | 0.1176 | 0.1438 |
| Day sleep | -0.0480 | 0.0785 | -0.0990 |
| Night bout number | -0.1703 | -0.1704 | **-0.2345** |
| Day bout number | 0.0297 | 0.0350 | **-0.3564** |
| Night avg. bout length | 0.1840 | **0.2276** | **0.2331** |
| Day avg. bout length | -0.1078 | 0.0272 | 0.0889 |
| Waking activity | -0.1551 | -0.0939 | 0.1060 |
| Night sleep *CV_E_* | -0.0330 | -0.0756 | -0.1607 |
| Day sleep *CV_E_* | 0.1031 | -0.0476 | 0.0835 |
| Night bout number *CV_E_* | **0.2427** | 0.1386 | **0.2317** |
| Day bout number *CV_E_* | 0.0419 | -0.0380 | 0.1502 |
| Night avg. bout length *CV_E_* | 0.0442 | -0.0760 | -0.0587 |
| Day avg. bout length *CV_E_* | -0.0615 | 0.0091 | 0.1219 |
| Waking activity *CV_E_* | 0.0409 | 0.0711 | -0.1304 |

B)

| Trait | MESA period | χ^2^period | Rhythmicity index |
| --- | --- | --- | --- |
| Night sleep | 0.1502 | 0.1407 | 0.1905 |
| Day sleep | -0.0707 | 0.0974 | -0.1361 |
| Night bout number | **-0.2469** | -0.2079 | **-0.3169** |
| Day bout number | 0.0466 | 0.0462 | **-0.5213** |
| Night avg. bout length | **0.2700** | **0.2810** | **0.3187** |
| Day avg. bout length | -0.2006 | 0.0426 | 0.1542 |
| Waking activity | -0.2173 | -0.1106 | 0.1383 |
| Night sleep *CV_E_* | -0.0485 | -0.0935 | -0.2201 |
| Day sleep *CV_E_* | 0.1681 | -0.0652 | 0.1269 |
| Night bout number *CV_E_* | **0.4142** | 0.1990 | **0.3685** |
| Day bout number *CV_E_* | 0.0849 | -0.0649 | **0.2841** |
| Night avg. bout length *CV_E_* | 0.0867 | -0.1253 | -0.1071 |
| Day avg. bout length *CV_E_* | -0.1408 | 0.0175 | **0.2603** |
| Waking activity *CV_E_* | 0.0631 | 0.0923 | -0.1875 |

C)

| Trait | MESA period | χ^2^ period | RI |
| --- | --- | --- | --- |
| Night sleep | 0.0546 | -0.0618 | 0.1007 |
| Day sleep | 0.0182 | 0.1955 | **-0.2585** |
| Night bout number | -0.1265 | -0.0816 | **-0.3591** |
| Day bout number | -0.1101 | -0.0010 | **-0.4276** |
| Night avg. bout length | 0.1454 | 0.0286 | 0.2092 |
| Day avg. bout length | 0.1395 | **0.2636** | 0.0301 |
| Waking activity | -0.0853 | -0.1188 | 0.2013 |

D)

| Trait | MESA period | χ^2^period | Rhythmicity index |
| --- | --- | --- | --- |
| Night sleep | 0.0865 | -0.0766 | 0.1392 |
| Day sleep | 0.0278 | **0.2351** | **-0.3465** |
| Night bout number | -0.2102 | -0.1062 | **-0.5213** |
| Day bout number | -0.1835 | -0.0013 | **-0.6227** |
| Night avg. bout length | **0.2257** | 0.0348 | **0.2837** |
| Day avg. bout length | 0.2155 | **0.3245** | 0.0414 |
| Waking activity | -0.1282 | -0.1403 | **0.2652** |
